# Gut microbial diversity at baseline conditions the clinical, microbiome, and metabolic response to paraprobiotic *Lactiplantibacillus plantarum* LRCC5282 in overweight adults

**DOI:** 10.1080/19490976.2026.2722835

**Published:** 2026-08-25

**Authors:** Ahyoung Lim, Sunghan Kim, Woongkwon Kwak, Husung Shim, Joongki Kwak, Seokmin Yoon

**Affiliations:** a Division of Product Application, LOTTE R&D Center, Seoul, Republic of Korea; b Division of Beverage, LOTTE R&D Center, Seoul, Republic of Korea

**Keywords:** Gut microbiota, paraprobiotic, *Lactiplantibacillus plantarum*, gut microbial diversity, obesity, randomized controlled trial

## Abstract

The gut microbiota is increasingly recognized as a target for obesity management; however, whether baseline gut microbial diversity conditions responsiveness to microbiota-targeted interventions remains unclear. We aimed to investigate whether baseline gut microbial diversity is associated with responsiveness to a paraprobiotic derived from *Lactiplantibacillus plantarum* LRCC5282 (LP5282-*P*) in overweight adults. In a 12-week, randomized, double-blind, placebo-controlled, multicenter trial of 120 overweight adults, LP5282-*P* produced no significant between-group differences in any clinical outcome across the overall per-protocol population. However, in the low-diversity subgroup, LP5282-*P* was associated with significant reductions in body weight, body mass index, and circulating leptin levels. These clinical changes were accompanied by compositional shifts in the gut microbiota, including higher relative abundances of *Christensenellaceae, Faecalibacterium*, and *Alistipes*. Fecal metabolite profiles showed elevated acetate and butyrate concentrations and altered bile acid composition. Within the low-diversity subgroup, changes in the relative abundances of *Akkermansia* and *Eubacterium* were inversely correlated with changes in body weight, body fat mass, and leptin levels. In contrast, the high-diversity subgroup exhibited no consistent response across the outcome domains examined. Overall, baseline gut microbial diversity was associated with differential responsiveness to LP5282-*P*, supporting its potential use as a stratification variable in future microbiota-targeted intervention trials. Further studies integrating direct measures of microbial activity and host response are warranted to elucidate the biological pathways underlying this diversity-dependent responsiveness.

**Trial registration:** Clinical Research Information Service (CRIS), KCT0008119.

## Introduction

The gut microbiota constitutes an ecologically complex, metabolically active community whose compositional and functional integrity is integral to host metabolic homeostasis. Its disruption has been implicated in the pathogenesis of obesity and its cardiometabolic sequelae.^1-3^ Obesity affects approximately 16% of adults worldwide and confers a substantial risk of insulin resistance, dyslipidemia, type 2 diabetes, and cardiovascular diseases.^4^ ^,^ ^5^ In individuals with obesity, characteristic shifts in microbiota composition, including an elevated *Firmicutes*-to-*Bacteroidota* (F/B) ratio, contraction of cytoprotective taxa such as *Akkermansia muciniphila* and *Faecalibacterium prausnitzii*, and expansion of *Proteobacteria*, collectively augment intestinal energy harvest, compromise epithelial barrier integrity, and drive metabolic endotoxemia-mediated chronic low-grade inflammation, thereby amplifying adipose expansion and metabolic deterioration.^6^ ^,^ ^7^ Predicated on this mechanistic framework, microbiota-targeted interventions spanning probiotics, prebiotics, synbiotics, postbiotics, and paraprobiotics have been extensively investigated as candidate strategies for obesity management.^8^ ^,^ ^9^ Systematic reviews and meta-analyses of randomized controlled trials have consistently reported modest population-level effect sizes alongside substantial inter-study heterogeneity in effect estimates.^8^ ^,^ ^10^ This heterogeneity cannot be explained solely by variations in study design or strain selection; instead, it suggests the presence of systematic, subject-level differences in responsiveness. Such differences are inherently difficult to capture using aggregate analysis.

A coherent account of inter-individual variability in obesity requires situating reduced *α*-diversity within its proper biological context. In a seminal metagenomic analysis of 292 adults, Le Chatelier et al.^7^ demonstrated that individuals with low bacterial gene richness exhibited markedly greater adiposity, insulin resistance, dyslipidemia, and systemic inflammation than their high-richness counterparts, and experienced disproportionate weight gain over time. This divergence does not merely reflect a quantitative difference in species number but instead represents a qualitative distinction in the functional state of the microbial community.^11^ Specifically, the depletion of key butyrate-producing genera, including *Faecalibacterium* and *Roseburia*, together with the contraction of bile acid-biotransforming taxa within *Ruminococcaceae* and *Lachnospiraceae*, led to reduced short-chain fatty acid (SCFA) production and the disruption of primary-to-secondary bile acid conversion. This disruption impairs FXR- and TGR5-mediated metabolic signaling.^12-14^ These metabolic alterations contribute to impaired intestinal barrier function, increased systemic lipopolysaccharide (LPS) translocation, and dysregulation of adipose lipid homeostasis. Collectively, these features define a state of community-level functional impairment that is qualitatively distinct from the high functional redundancy and ecological resilience observed in gene-rich microbiota.^15^ Thus, within obese populations, *α*-diversity reflects not merely species richness but the extent of functional and ecological impairment. This delineates the microbial community states with differential capacities to respond to external perturbations, warranting direct investigation.

The divergent functional states of microbial communities give rise to differential responses to exogenous stimuli, as supported by converging evidence from ecological theories and human intervention studies. Highly diverse microbial communities densely occupy metabolic niches and maintain elevated functional redundancy, properties that confer structural stability and colonization resistance against external perturbations.^11^ Spragge et al.^16^ provided direct experimental validation of this principle in a gut microbial context, demonstrating that community-level colonization resistance scales nonlinearly with diversity through competitive nutrient blockade. Human intervention studies extended these ecological predictions to include clinically observable outcomes. By integrating eight independent dietary intervention datasets, Klimenko et al.^17^ identified a reproducible inverse relationship between baseline *α*-diversity and the magnitude compositional change in the microbiota, independent of intervention type. In a prospective randomized dietary trial, Wastyk et al.^18^ showed that immune response trajectories to a high-fiber intervention diverged according to the baseline microbiota diversity. Similarly, Zmora et al.^19^ demonstrated that inter-individual variations in probiotic colonization and their downstream effects on community structure are governed by pre-existing host and microbiome characteristics. Collectively, these findings indicate that high baseline *α*-diversity buffers microbial communities against intervention-induced restructuring. In contrast, low-diversity communities undergo extensive and directionally consistent reorganization under equivalent stimuli.^11^ ^,^ ^20^ This positions baseline *α*-diversity as a key ecological determinant of variability in responsiveness to microbiota-targeted interventions. Extending this framework to paraprobiotics administered to treat obesity-associated dysbiosis is clinically relevant, as baseline gut microbial diversity may represent a reproducible stratification marker for paraprobiotic responsiveness. However, this proposition has not been comprehensively evaluated in controlled trial settings, and additional evidence is needed to substantiate its utility as a mechanistic framework for interpreting inter-individual heterogeneity and informing microbiome diversity profiling in future trial designs.

The present study aimed to evaluate whether baseline gut microbial *α*-diversity conditions the clinical, microbiome, and fecal metabolite responses to a paraprobiotic derived from *Lactiplantibacillus plantarum* LRCC5282 (LP5282-*P*) in overweight adults. LP5282-*P* has previously demonstrated anti-obesity efficacy through gut microbiota modulation in high-fat diet-induced murine models and 3T3-L1 adipocytes (referred to as postbiotics in the original publication).^21^ The intervention was evaluated in a 12-week, randomized, double-blind, placebo-controlled, multicenter trial in overweight adults. Clinical efficacy was first assessed across the entire per-protocol population; participants were then stratified by baseline gut microbial *α*-diversity to examine differential responses across clinical, microbiome, and metabolite outcomes. This approach was intended to clarify inter-individual variability in responsiveness to microbiota-targeted interventions and to inform the integration of microbiome diversity profiling into future intervention studies.

## Materials and Methods

### Study design

This 12-week, randomized, double-blind, placebo-controlled, parallel-group clinical trial was conducted at three sites.^22^ The 12-week intervention period was prespecified on the basis of regulatory and methodological considerations. A minimum duration of 12 weeks is recommended for evaluating body fat–reduction efficacy in clinical trials of functional foods,^23^ and this duration has been commonly adopted in randomized controlled trials of microbiota-targeted interventions that report body composition outcomes in adults with overweight or obesity.^8^ ^,^ ^24^ ^,^ ^25^ Participants were randomly assigned in a 1:1 ratio to the LP5282-*P* or placebo group using center-stratified block randomization. Allocation sequences were generated using the SAS randomization program (version 9.4; SAS Institute Inc., Cary, NC, U.S.A.). Treatment codes were then applied sequentially to pre-labeled investigational products according to the site-specific participant identification numbers. Sealed allocation envelopes were retained by a designated individual within the sponsor organization who had no involvement in conducting the trial. Blinding was maintained for participants, investigators, and the contract research organization throughout the intervention period. The contract research organization performed unblinding after the per-protocol set was finalized. The concomitant use of medications, health supplements, or structured weight-control programs was restricted during the intervention period unless deemed unavoidable and reported to the investigators. The study consisted of four visits: visit 1 corresponded to screening, visit 2 to day 0 (randomization), visit 3 to day 42 ± 7 (week 6), and visit 4 to day 84 ± 5 (week 12).

### Participants

A target sample size of 120 participants (*n* = 60 per group) was determined based on the primary endpoint, which was defined as the change in total body fat mass from baseline to week 12. Based on a previous 12-week randomized controlled trial reporting a between-group difference in total body fat mass of 1.5 kg with a pooled standard deviation of 2.27 kg,^26^ a minimum of 50 participants per group were required to detect this difference at a two-sided significance level of 0.05 with 90% power. The total target sample size (120 participants) also accounted for an anticipated dropout rate of 20%. The participants were recruited between April 2022 and September 2022 across three clinical sites. These sites included St. Vincent's Hospital at the Catholic University of Korea, Hallym University Sacred Heart Hospital, and Seoul-Boramae Hospital. The follow-up was completed in September 2022. The trial was conducted by Neonutra Co., Ltd., a contract research organization commissioned by the study sponsor, LOTTE Chilsung Beverage Co., Ltd.

Eligible participants were men and women aged 19–65 years with a body mass index (BMI) between 25 and 30 kg/m^2^ at both screening and baseline visits. This BMI range corresponds to grade 1 obesity as defined by the Korean Society for the Study of Obesity clinical guidelines.^27^ ^,^ ^28^ The major exclusion criterion was a history of severe cardiovascular, hepatic, renal, endocrine, or psychiatric disorders. Additional exclusion criteria included active malignancy requiring treatment and the use of medications or supplements known to affect body weight or gut microbiota composition within the preceding 2–4 weeks. These include anti-obesity agents, antidepressants, corticosteroids, probiotics, prebiotics, and bowel-regulating agents. Participants were also excluded if they had a history of obesity-related surgery within the past year, uncontrolled hypertension defined as systolic and diastolic blood pressure ≥160/100 mmHg, fasting plasma glucose ≥126 mg/dL, or were receiving treatment for diabetes. All participants provided written informed consent before participation.

### Paraprobiotic investigational product and intervention

The investigational product was formulated using paraprobiotics derived from *L. plantarum* LRCC5282 (LP5282-P; LRCC, LOTTE R&D Culture Collection), a strain originally isolated from Korean fermented foods and previously characterized in preclinical models of diet-induced obesity.^21^ The strain was developed and maintained at the LOTTE R&D Center (Seoul, Republic of Korea). The final investigational product was manufactured under a contract with Lactomason Co., Ltd. (Jinju, Republic of Korea), a facility certified under the Good Manufacturing Practice standards for health functional foods. The LP5282-derived paraprobiotic material was prepared as previously described.^21^ Briefly, LP5282 was cultivated under controlled fermentation conditions and thermally inactivated at 90 °C for 30 min. The material was freeze-dried to obtain a paraprobiotic powder (LP5282-*P*). Cell viability was quantified before and after heat treatment to confirm complete thermal inactivation. Three independent manufacturing batches were produced, and inter-batch consistency was confirmed prior to clinical use. The resulting LP5282-*P* powder contained more than 2 × 10^11^ cells per gram and was standardized by blending with maltodextrin to ensure that each packet provided a defined cell equivalent of 1.46 × 10^10^ cells in 1,500 mg of LP5282-*P*. The final investigational product was formulated as single-dose packets (1,600 mg). Each packet contained 1,500 mg standardized LP5282-*P*, 84 mg maltodextrin, and 16 mg silicon dioxide. The placebo products consisted of maltodextrin and silicon dioxide without LP5282-*P*, and were identical in appearance, weight, and packaging as that of the interventional product. The participants in the investigational group received LP5282-*P*, whereas those in the placebo group received an identically formulated placebo. All participants consumed a single 1,600-mg packet once daily for 12 weeks. To minimize the potential variability arising from lifestyle-related factors, all participants received standardized, non-prescriptive guidance aimed at maintaining consistent dietary and physical activity patterns throughout the intervention period. No supervised or structured lifestyle interventions were implemented. Dietary intake and physical activity were assessed at baseline, week 6, and week 12. At each visit, participants completed a dietary record covering the preceding three days to monitor dietary patterns and detect any extreme dietary changes, including fasting or overeating.^26^ Physical activity was assessed using the Global Physical Activity Questionnaire.^29^ Domain-specific physical activity (work, travel, and recreation), total activity, and sedentary time were derived from the questionnaire and expressed as both duration and metabolic equivalent of task (MET)-based estimates.

## Ethical approval and trial registration

This trial was conducted in accordance with the Declaration of Helsinki and the International Conference on Harmonization-Good Clinical Practice guidelines.^30^ ^,^ ^31^ All study procedures were reviewed and approved by the Institutional Review Boards of the following participating institutions: St. Vincent's Hospital of the Catholic University of Korea (VC22HDDE0007), Hallym University Sacred Heart Hospital (HALLYM 2022-01-018), and Seoul-Boramae Hospital (20-2022-10). All participants provided written informed consent before participation. The study was registered with the Clinical Research Information Service (CRIS; KCT0008119; https://cris.nih.go.kr/cris/search/detailSearch.do?seq=23380&search_page=L). No protocol amendments were made after trial registration, and no protocol updates occurred prior to data analysis. Preventive safety measures were implemented throughout the trial and adverse events were monitored at each visit. All participants were covered by clinical trial insurance (Lotte Insurance Co., Ltd., Seoul, Republic of Korea), which ensured compensation for study-related injuries. The full trial protocol is not publicly available but can be obtained from the corresponding author upon reasonable request.

### Analysis populations

The analysis populations were predefined before database lock and unblinding. The full analysis (FA) set included all randomized participants who received at least one dose of the investigational product and had at least one post-baseline efficacy assessment, consistent with the intention-to-treat principle.^32^ The per-protocol (PP) set comprised participants from the FA population who completed the 12-week intervention without major protocol deviations that could affect the efficacy evaluation. Major protocol deviations included the use of prohibited concomitant medications, meaningful deviations from the scheduled visit timeline, or the occurrence of intercurrent medical conditions likely to influence metabolic outcomes. Participants with insufficient compliance, defined as the consumption of less than 80% of the prescribed investigational product, were excluded from the PP analysis.^32^ Compliance was assessed by counting the sachets returned at each visit and expressed as the proportion of consumed sachets relative to the expected number. All determinations regarding the inclusion of the analysis set were finalized prior to unblinding. Efficacy analyses were conducted on the PP set, and the FA set was retained for supportive analysis. Stratified analyses within the PP set were based on a composite baseline *α*-diversity score. Baseline diversity has been associated with intervention responsiveness and used to stratify participants into high- and low-diversity groups.^7^ ^,^ ^17^ ^,^ ^33^ In the present study, the composite score integrated multiple baseline *α*-diversity indices. Within each treatment group, each index was converted to a percentile rank and averaged across indices to yield a composite score per participant. Participants were then ranked by this score and divided at the group median into high- and low-diversity subgroups, with values at or above the median classified as high. Two subgroups, rather than tertiles or quartiles, were used to retain adequate subgroup sizes for the within-subgroup clinical, compositional, and metabolite analyses after division by treatment assignment. The clinical outcomes, gut microbiota composition, and fecal metabolite profiles were assessed within each subgroup.

### Clinical outcomes and anthropometric assessments

Clinical outcomes included changes in body fat mass assessed using dual-energy X-ray absorptiometry (DXA); anthropometric indices, including body weight, BMI, waist circumference, hip circumference, and waist-to-hip ratio; and abdominal adiposity parameters, including visceral fat area, subcutaneous fat area, total abdominal fat area, and visceral-to-subcutaneous fat ratio. Body composition was measured using a DXA densitometer (Lunar Prodigy; GE Healthcare, Madison, WI, U.S.A.). Abdominal fat distribution was assessed using computed tomography at the L4–L5 intervertebral level (Somatom Definition AS, Siemens Healthineers, Germany), and adipose tissue areas were quantified using FatScan software (N2 System, Osaka, Japan) based on attenuation thresholds ranging from −190 to −30 Hounsfield units.^34^ Anthropometric measurements were obtained in triplicate by trained personnel using standardized procedures: body weight was measured to the nearest 0.1 kg and height to the nearest 0.1 cm, and waist and hip circumferences were measured using a non-elastic tape. Assessments were conducted at baseline and at week 12, and additional anthropometric measurements were performed at week 6. These clinical and anthropometric parameters were evaluated in both the overall PP population and the diversity-stratified subgroups. No changes to the pre-specified clinical outcome measures were made after trial commencement.^22^

### Blood biochemical analyses

Fasting venous blood samples were collected following an overnight fast of at least 8 hours at baseline, week 6, and week 12. Blood was allowed to clot at 25 °C and centrifuged to obtain the serum, which was used for all biochemical measurements. Serum total cholesterol, triglycerides, high-density lipoprotein (HDL) cholesterol, low-density lipoprotein (LDL) cholesterol, and high-sensitivity C-reactive protein (hs-CRP) levels were quantified using an automated chemistry analyzer (Hitachi 7600-210, Hitachi High-Tech, Japan). The analyzer was calibrated according to the manufacturer's specifications and verified using internal quality control procedures. Fasting serum glucose, renal function markers (blood urea nitrogen and creatinine), albumin, total protein, total bilirubin, and uric acid levels were measured using the same analyzer under identical quality control procedures. Liver function was assessed by measuring aspartate aminotransferase (AST), alanine aminotransferase (ALT), and *γ*-glutamyl transferase (GGT). Adiponectin and leptin concentrations were measured at an accredited central laboratory (GCCL, Yongin, Republic of Korea) operating under Good Clinical Laboratory Practice standards, using commercial ELISA kits (R&D Systems, Minneapolis, MN, U.S.A.) according to the manufacturer's protocol. All assays were performed in duplicate with internal quality controls. These biochemical parameters were assessed in the overall PP population and within the diversity-stratified subgroups.

### Fecal sample collection and microbiome profiling

Fecal samples for gut microbiota profiling, a prespecified secondary efficacy outcome, were collected using a room-temperature-stable collection kit (TheBiome®, Macrogen Inc., Seoul, Republic of Korea) either on the day of the study visit or within 1–2 days prior. Samples were handled and transported according to the manufacturer's instructions and stored at −80 °C until analysis. Microbial genomic DNA was extracted from approximately 200 mg of fecal material using a FastDNA Spin Kit for Soil (MP Biomedicals, CA, U.S.A.). The V3–V4 region of the bacterial 16S rRNA gene was amplified using the primers 341F and 805R. Paired-end sequencing (2 × 300 bp) was performed using an Illumina MiSeq platform (Macrogen Inc., Seoul, Republic of Korea). Sequence data were processed using the DADA2 pipeline, including quality filtering, denoising, chimera removal, and inference of amplicon sequence variants (ASVs).^35^ Taxonomic assignments were performed using the SILVA reference database.^36^ *α*-diversity indices, including observed operational taxonomic units (OTUs), as well as the Shannon diversity, Simpson, Chao1, ACE, and inverse Simpson indices, were calculated using the phyloseq R package.^37^ *α*-diversity was calculated at baseline to characterize gut microbial diversity at the time of randomization and to support within-group stratification by diversity status. *β*-diversity was assessed using the Bray–Curtis dissimilarity test. Group-level differences in community structure were evaluated using PERMANOVA, with 999 permutations implemented using the Adonis function in the R package vegan.^38^ The functional profiles of the gut microbiota were inferred using PICRUSt2, based on amplicon sequence variant (ASV)-level taxonomic assignments. The predicted gene family abundances were mapped to Kyoto Encyclopedia of Genes and Genomes (KEGG) orthologs (KOs) for pathway-level interpretation.^39^ Functional profiles inferred using PICRUSt2 are based on computational predictions and should be interpreted with caution, as they do not represent experimentally validated metabolic activities or causal host effects.^40^

### Fecal metabolite analysis

Fecal concentrations of SCFAs and bile acids were measured at baseline and at week 12 as indicators of gut microbial metabolic activity. SCFAs, including acetate, propionate, and butyrate, were quantified via high-performance liquid chromatography using an Ultimate 3000 system (Thermo Fisher Scientific, Waltham, MA, U.S.A.) equipped with an Aminex HPX-87H column (300 × 10 mm; Bio-Rad Laboratories, Hercules, CA, U.S.A.).^41^ Briefly, the samples were homogenized in distilled water and centrifuged at 12,000 × *g* for 20 min at 4 °C, and the resulting supernatants were injected into the column. Separation was achieved using 0.01 *N* sulfuric acid as the mobile phase at a flow rate of 0.5 mL/min. The analytes were detected using a refractive index detector (RefractoMAX520, ERC, Japan), and quantification was performed against external calibration curves. Seven bile acid species were quantified using liquid chromatography-tandem mass spectrometry. These comprised the primary bile acids cholic acid (CA) and chenodeoxycholic acid (CDCA), the secondary bile acids deoxycholic acid (DCA), lithocholic acid (LCA), and ursodeoxycholic acid (UDCA), and the conjugated bile acids glycocholic acid (GCA) and taurocholic acid (TCA). Stable isotope-labeled internal standards (CA-d4, CDCA-d4, DCA-d4, LCA-d4, UDCA-d4, GCA-d4, and TCA-d4; Sigma-Aldrich, St. Louis, MO, U.S.A.) were added to each sample before extraction.^42^ Chromatographic separation was achieved using a Thermo Hypersil Gold C18 column with gradient elution. Detection was performed in the negative electrospray ionization mode using selected ion monitoring for unconjugated bile acids and multiple reaction monitoring for conjugated species. The instrument parameters were set as follows: ion spray voltage, −4500 V; source temperature, 450 °C; curtain gas, 30.0 psi; and ion source gas pressures of 40 and 50 psi. Quantification was performed via stable isotope dilution using external calibration curves, as previously described.^43^

### Differential abundance and correlation analyses

Differentially abundant taxa were identified using the sequential analytical approach. Linear discriminant analysis effect size (LEfSe) was used as an exploratory screening step to identify the candidate taxa that contribute to group separation.^44^ Analysis of composition of microbiomes with bias correction (ANCOM-BC) was subsequently used for primary differential abundance analysis to account for compositional bias in the microbiome count data.^45^ The principal comparisons were LP-L vs. PL-L at week 12 and LP-H vs. PL-H at week 12. Within-group longitudinal comparisons between the baseline and at week 12 were performed for each subgroup. Multivariable association analysis was performed using MaAsLin2, with age, sex, and baseline BMI as covariates. This analysis evaluated whether the taxonomic associations with LP5282-*P* administration were independent of baseline host characteristics.^46^ The concordance of the findings across the three methods was used to assess the robustness of the results. Spearman’s correlation analyseswere performed to examine the association between changes in relative microbial abundance and changes in fecal metabolite concentrations, including butyrate and bile acid species. Associations between metabolite changes and clinical parameters, including body fat mass and leptin levels, were also evaluated. These analyses were conducted within the LP-L subgroup, using the PL-L and LP-H groups as comparative references. All *p*-values were adjusted for multiple comparisons using the Benjamini–Hochberg false discovery rate correction separately applied within each analytical framework.^47^

### Statistical analysis

Statistical analyses were performed using GraphPad Prism version 10.6.1 (GraphPad Software, San Diego, CA, U.S.A.) and the R statistical software. Data are presented as the mean ± standard error of the mean. Between-group comparisons were conducted using parametric or nonparametric tests based on the Shapiro–Wilk normality assessment. The endpoint was analyzed using analysis of covariance (ANCOVA), with baseline values as covariates, and the treatment effects were reported with 95% confidence intervals. ANCOVA was also applied to the outcomes for which baseline imbalances were observed. For the stratified analyses, treatment-by-diversity subgroup interactions were evaluated using a linear model that incorporated the treatment group, diversity subgroup, and their interaction terms as independent variables. All statistical tests were two-sided, with a significance threshold of *p* < 0.05. *P*-values were adjusted for multiple comparisons using the Benjamini–Hochberg false discovery rate correction. Dietary intake and physical activity were not included as covariates in the models for clinical outcomes or the taxonomic association models, and neither was tested for association with baseline diversity or treatment response. As dietary intake was assessed only qualitatively, quantitative analysis of this variable was not possible. For additional confirmation, physical activity was evaluated as a covariate in a post hoc analysis of the four subgroups.

## Results

### Participant flow and analysis populations

**Figure 1.**
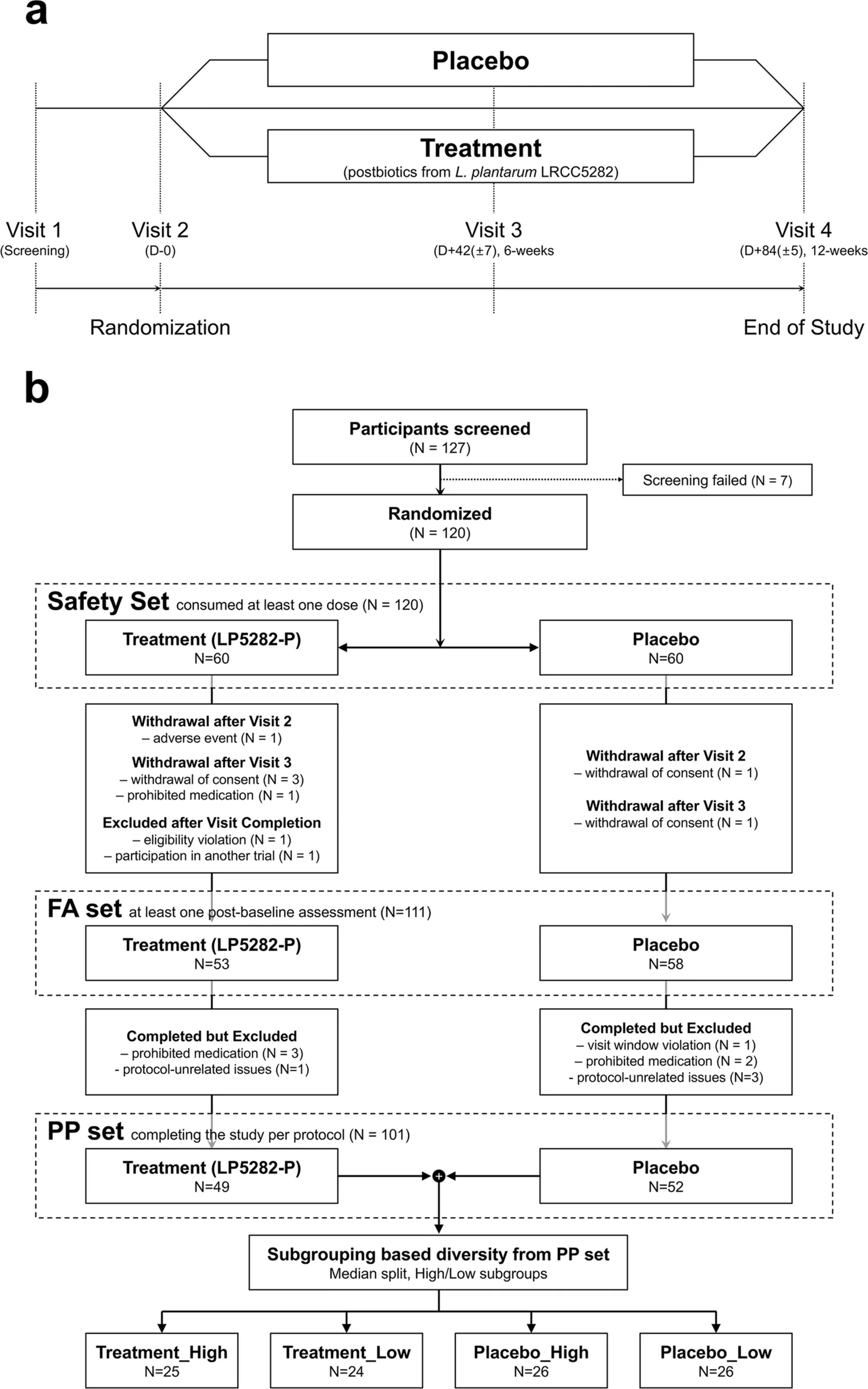
Study design and participant flow. (a) Study design and visit schedule. Participants were randomized at baseline (visit 2, Day 0) to receive a paraprobiotic preparation derived from *Lactiplantibacillus plantarum* LRCC5282 (LP5282-*P*) or placebo for 12 weeks, with follow-up visits conducted at week 6 (visit 3; Day 42 ± 7) and week 12 (visit 4; Day 84 ± 5). (b) CONSORT-style flow diagram illustrating participant screening, randomization, and derivation of the Safety set, full analysis (FA) set, and per-protocol (PP) set, with reasons for exclusion indicated at each stage. Within the PP set, participants were stratified into high- and low-diversity subgroups by median split of a composite baseline gut microbial *α*-diversity score within each treatment group, yielding four subgroups: LP-H (*n* = 25), LP-L (*n* = 24), PL-H (*n* = 26), and PL-L (*n* = 26). FA, full analysis set; PP, per-protocol set; LP-H, high-diversity LP5282-*P* subgroup; LP-L, low-diversity LP5282-*P* subgroup; PL-H, high-diversity placebo subgroup; PL-L, low-diversity placebo subgroup.

The study design and visit schedule are shown in Figure 1a. A total of 127 individuals were screened, of whom, 7 were excluded owing to ineligibility. The remaining 120 participants received at least one dose of the assigned intervention and constituted the safety set, with 60 participants randomized to each group. No serious adverse events were reported during the intervention period and the incidence of adverse events did not differ between the two groups. Among the 120 participants in the safety set, 9 were excluded from the FA set owing to the absence of post-baseline efficacy assessments (*n* = 7), post-enrollment identification of eligibility violations (*n* = 1), or participation in another clinical trial (*n* = 1), yielding an FA set of 111 participants (LP5282-*P*, *n* = 53; placebo, *n* = 58). The PP set comprised participants from the FA population who completed the 12-week intervention without major protocol deviations likely to affect the efficacy evaluation. Ten participants were excluded because of prohibited concomitant medication use (*n* = 5), intercurrent COVID-19 infection associated with a substantial lifestyle or metabolic disruption (*n* = 4), or noncompliance with the predefined visit window (*n* = 1). This resulted in a final PP set of 101 participants (LP5282-*P*, *n* = 49; placebo, *n* = 52; Figure 1b).

### Overall clinical and microbiome outcomes in the PP population

The investigated characteristics were comparable between the LP5282-*P* group at baseline and placebo groups at baseline (LP-BL and PL-BL, respectively), with no statistically significant between-group differences observed (Supplementary Table 1). After the 12-week intervention, no statistically significant between-group differences were observed in any clinical outcomes in the LP5282-*P* group at week 12 (LP-12) or the placebo group at week 12 (PL-12) (Table 1). The DXA-derived total body fat mass decreased in both groups, with no significant between-group differences. Body weight decreased by −1.33 ± 0.28 kg in LP-12 compared with LP-BL, and −0.94 ± 0.26 kg in PL-12 compared with PL-BL; this difference did not reach statistical significance. In addition, the serum lipid parameters, adipokine levels (including leptin and adiponectin), and abdominal adiposity indices showed no significant between-group differences. The biochemical safety markers were also similar between groups (Supplementary Table 2).

**Table 1.** Changes in anthropometric, body composition, and metabolic parameters at baseline and week 12 in the per-protocol population.

Parameters	Placebo (*n* = 52)	LP5282-P (*n* = 49)	*P*-value
Body weight (kg)			
- visit 2 (baseline)	72.84 ± 1.12	73.50 ± 1.26	0.6974
- visit 4 (after 12 weeks)	71.74 ± 1.20	72.22 ± 1.23	0.7808
- changes from baseline	−0.94 ± 0.26	−1.33 ± 0.28	0.3165
BMI (kg/m^2^)			
- visit 2 (baseline)	27.13 ± 0.19	26.80 ± 0.20	0.2240
- visit 4 (after 12 weeks)	26.71 ± 0.20	26.32 ± 0.20	0.1802
- changes from baseline	−0.38 ± 0.10	−0.49 ± 0.10	0.4448
DXA (fat mass, g)			
- visit 2 (baseline)	25,563.00 ± 611.8	25,712.16 ± 590.6	0.8614
- visit 4 (after 12 weeks)	24,992.88 ± 561.1	25,133.92 ± 587.6	0.8625
- changes from baseline	−570.1 ± 201.3	−578.2 ± 231.7	0.9789
DXA (fat mass, %)			
- visit 2 (baseline)	36.76 ± 0.97	36.64 ± 0.90	0.9276
- visit 4 (after 12 weeks)	36.43 ± 0.93	36.28 ± 0.92	0.9086
- changes from baseline	−0.32 ± 0.21	−0.35 ± 0.25	0.9269
DXA (lean, g)			
- visit 2 (baseline)	44,732 ± 1,238	45,153 ± 1,243	0.8108
- visit 4 (after 12 weeks)	44,449.37 ± 1,278	44,803.33 ± 1,221.83	0.8421
- changes from baseline	−282.7 ± 161.4	−349.67 ± 175.1	0.7773
Total Cholesterol (mg/dL)			
- visit 2 (baseline)	210.0 ± 5.17	200.6 ± 4.72	0.1820
- visit 4 (after 12 weeks)	201.9 ± 4.71	198.9 ± 4.95	0.6625
- changes from baseline	−8.15 ± 3.29	−1.69 ± 4.21	0.2264
HDL-Cholesterol (mg/dL)			
- visit 2 (baseline)	54.92 ± 1.61	54.14 ± 1.68	0.7379
- visit 4 (after 12 weeks)	55.21 ± 1.75	55.12 ± 1.69	0.9709
- changes from baseline	0.29 ± 0.81	0.98 ± 0.86	0.5587
LDL-Cholesterol (mg/dL)			
- visit 2 (baseline)	129.9 ± 4.79	123.9 ± 4.42	0.3576
- visit 4 (after 12 weeks)	125.5 ± 3.79	120.2 ± 5.26	0.4136
- changes from baseline	−4.44 ± 3.30	−3.67 ± 4.69	0.8926
Triglycerides (mg/dL)			
- visit 2 (baseline)	133.2 ± 8.68	127.4 ± 10.60	0.6712
- visit 4 (after 12 weeks)	131.3 ± 8.81	123.3 ± 12.09	0.5899
- changes from baseline	−1.90 ± 7.58	−4.12 ± 6.91	0.8298
hs-CRP (mg/dL)			
- visit 2 (baseline)	3.09 ± 0.77	3.76 ± 1.38	0.6690
- visit 4 (after 12 weeks)	4.66 ± 1.34	4.12 ± 1.80	0.8075
- changes from baseline	1.57 ± 1.09	0.35 ± 0.55	0.3310
Adiponectin (μg/mL)			
- visit 2 (baseline)	5.30 ± 0.34	5.50 ± 0.30	0.6774
- visit 4 (after 12 weeks)	5.13 ± 0.33	5.58 ± 0.32	0.3328
- changes from baseline	−0.18 ± 0.17	0.08 ± 0.18	0.2839
Leptin (ng/mL)			
- visit 2 (baseline)	13.54 ± 1.14	11.69 ± 1.10	0.2466
- visit 4 (after 12 weeks)	12.39 ± 1.12	10.72 ± 1.05	0.2817
- changes from baseline	−1.15 ± 0.78	−0.97 ± 0.78	0.8689
Visceral fat area (cm^2^)			
- visit 2 (baseline)	120.7 ± 6.15	115.7 ± 5.67	0.5511
- visit 4 (after 12 weeks)	110.6 ± 5.88	108.0 ± 5.33	0.7445
- changes from baseline	−10.10 ± 3.72	−7.69 ± 4.10	0.6632
Subcutaneous fat area (cm^2^)			
- visit 2 (baseline)	253.7 ± 9.70	255.2 ± 10.39	0.9193
- visit 4 (after 12 weeks)	250.0 ± 9.74	242.7 ± 10.57	0.6115
- changes from baseline	−3.74 ± 3.55	−12.49 ± 5.92	0.2013
Total abdominal fat area (cm^2^)			
- visit 2 (baseline)	374.5 ± 10.53	370.9 ± 10.83	0.8135
- visit 4 (after 12 weeks)	360.6 ± 10.32	350.7 ± 10.48	0.5021
- changes from baseline	−13.84 ± 5.92	−20.18 ± 7.26	0.4977
Ratio of visceral/subcutaneous fat			
- visit 2 (baseline)	0.52 ± 0.04	0.50 ± 0.04	0.6389
- visit 4 (after 12 weeks)	0.49 ± 0.04	0.50 ± 0.04	0.8768
- changes from baseline	−0.04 ± 0.02	−0.00 ± 0.02	0.2182

BMI, body mass index; DXA, dual-energy X-ray absorptiometry; HDL, high-density lipoprotein; LDL, low-density lipoprotein; hs-CRP, high-sensitivity C-reactive protein; Placebo, placebo-treated group; LP5282-P, paraprobiotics derived from *Lactiplantibacillus plantarum* LRCC5282; n, number of participants included in the per-protocol analysis from each group. Data are presented as the mean ± SEM. *P*-values represent between-group comparisons, with P < 0.05 considered statistically significant.

**Figure 2.**
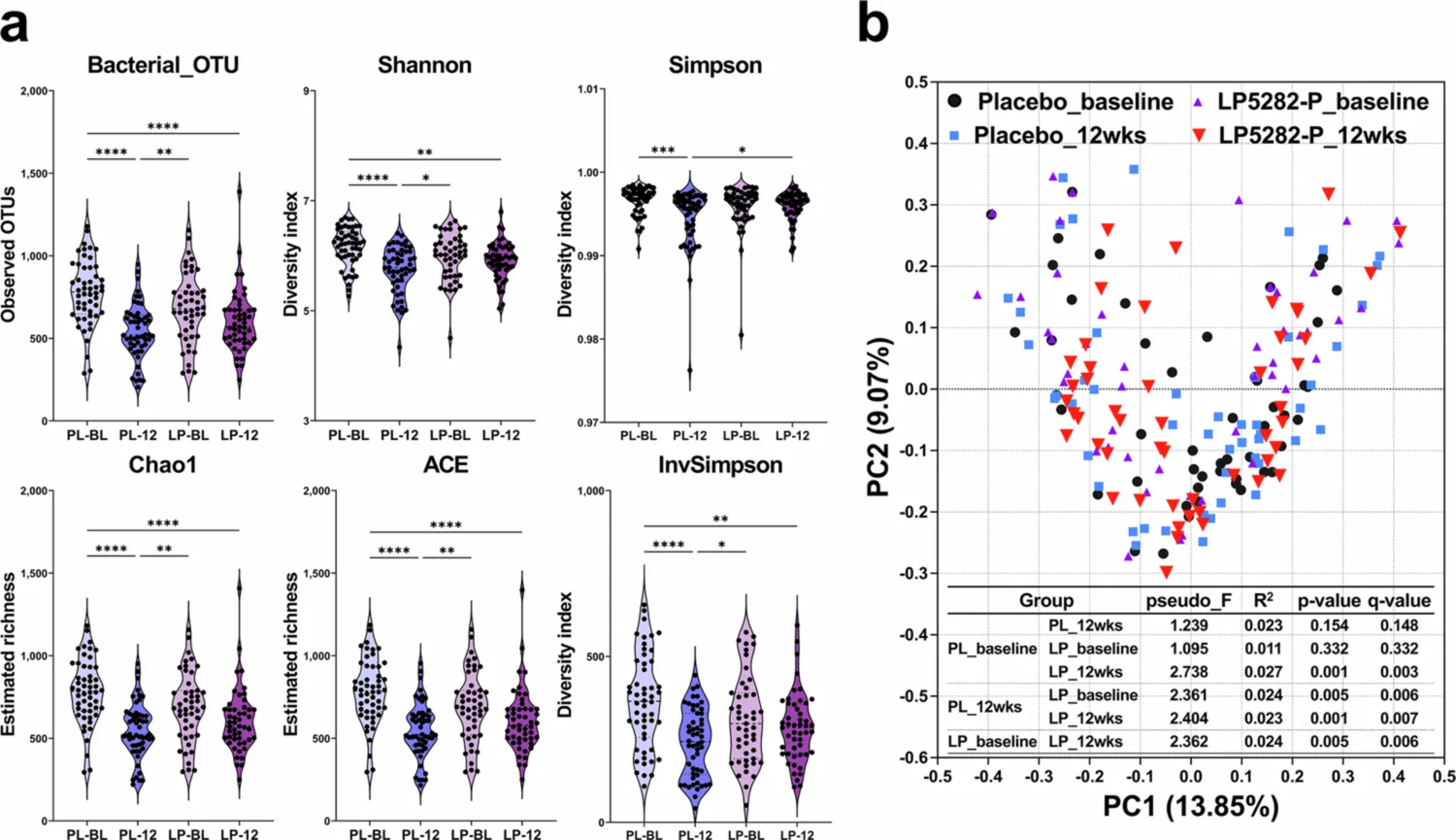
Gut microbiota diversity in the overall per-protocol population. (a) *α*-diversity indices, including observed OTUs, Shannon, Simpson, Chao1, ACE, and inverse Simpson, at baseline and week 12 in the LP5282-*P* and placebo groups. (b) Principal coordinates analysis (PCoA) based on Bray–Curtis dissimilarity at the genus level, with PERMANOVA statistics including pseudo-F, R^2^, *p*-value, and q-value shown in the inset table. PL-BL, placebo group at baseline; PL-12, placebo group at week 12; LP-BL, LP5282-*P* group at baseline; LP-12, LP5282-*P* group at week 12. LP5282-*P*, paraprobiotic derived from *Lactiplantibacillus plantarum* LRCC5282. PCoA, principal coordinates analysis; PC1 and PC2, first and second principal coordinates. q-values represent *p*-values adjusted for multiple comparisons using the Benjamini–Hochberg false discovery rate correction. Asterisks indicate statistical significance: *p < 0.05; **p < 0.01; ***p < 0.001; ****p < 0.0001.

The gut microbial *α*-diversity showed no significant between-group differences at week 12, although within-group increases were observed across all measured indices in the LP5282-*P* group over the intervention period (Figure 2a). *β*-diversity analysis based on Bray–Curtis dissimilarity revealed a significant separation of LP-12 from both PL-12 (pseudo-F = 2.40, R^2^ = 0.023, *p* = 0.001) and LP-BL (pseudo-F = 2.36, R^2^ = 0.024, *p* = 0.005), with no corresponding separation detected within the placebo group over time (Figure 2b). The absence of uniform between-group clinical effects, in conjunction with LP5282-*P*-associated microbiome restructuring, informed stratified analyses based on baseline gut microbial *α*-diversity.

### Diversity-stratified subgrouping and baseline characteristics

The baseline gut microbial *α*-diversity across the PP set was characterized using five indices (observed OTUs, Shannon, Simpson, Chao1, and ACE), which were converted to percentile ranks within each treatment group and averaged to yield a composite diversity score per participant. The participants in each treatment group were ranked using this composite score and divided into high- and low-diversity subgroups via a median split. Participants with scores at or above the median were assigned to the high-diversity subgroup, whereas those below the median were assigned to the low-diversity subgroup. The odd number of participants in the LP5282-*P* group (*n* = 49) resulted in a slightly different subgroup size. The resulting four subgroups comprised a high-diversity LP5282-*P* group (LP-H, *n* = 25), low-diversity LP5282-*P* group (LP-L, *n* = 24), high-diversity placebo group (PL-H, *n* = 26), and low-diversity placebo group (PL-L, *n* = 26; Figure 1b). The baseline characteristics within each subgroup were comparable between the LP5282-*P* and placebo groups, with no statistically significant between-group differences observed for any of the measured variables including age, sex, body weight, BMI, lifestyle factors, comorbid conditions, concomitant medication use, or investigational product compliance (Supplementary Tables 3 and 4). The biochemical safety markers were also comparable between the groups (Supplementary Tables 5 and 6).

### Clinical and microbiome outcomes in the diversity-stratified subgroups

**Figure 3.**
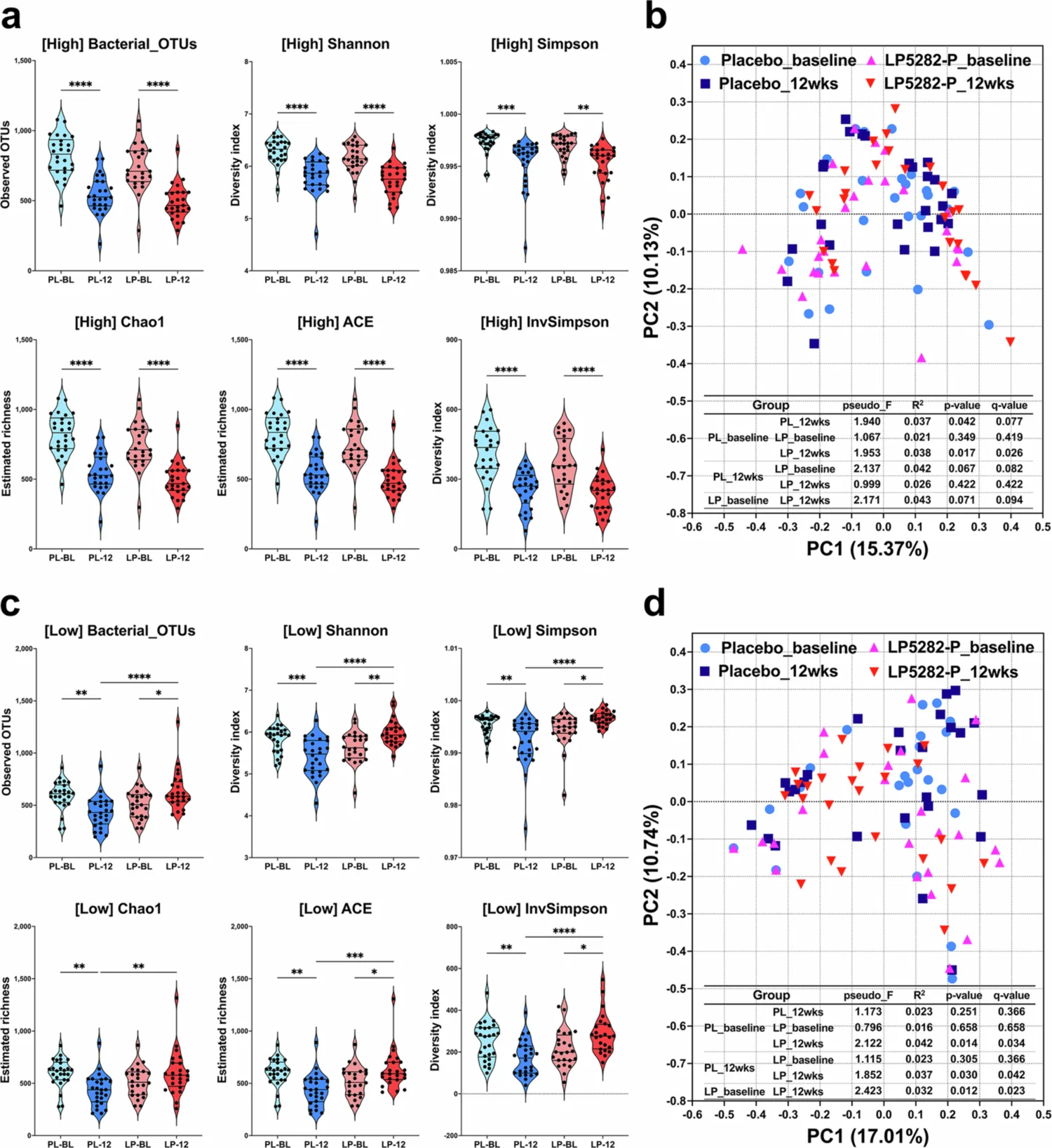
Gut microbiota diversity in diversity-stratified subgroups. (a) *α*-diversity indices, including observed OTUs, Shannon, Simpson, Chao1, ACE, and inverse Simpson, at baseline and week 12 in the high-diversity subgroup. (b) PCoA based on Bray–Curtis dissimilarity at the genus level in the high-diversity subgroup, with PERMANOVA statistics including pseudo-F, R^2^, *p*-value, and q-value shown in the inset table. (c) *α*-diversity indices at baseline and week 12 in the low-diversity subgroup. (d) PCoA based on Bray–Curtis dissimilarity at the genus level in the low-diversity subgroup, with PERMANOVA statistics shown in the inset table. PL-BL, placebo group at baseline; PL-12, placebo group at week 12; LP-BL, LP5282-*P* group at baseline; LP-12, LP5282-*P* group at week 12. LP5282-*P*, paraprobiotic derived from *Lactiplantibacillus plantarum* LRCC5282. PCoA, principal coordinates analysis; PC1 and PC2, first and second principal coordinates. q-values represent *p*-values adjusted for multiple comparisons using the Benjamini–Hochberg false discovery rate correction. Asterisks indicate statistical significance: *p < 0.05; **p < 0.01; ***p < 0.001; ****p < 0.0001.

In the high-diversity subgroup, LP5282-*P* administration did not elicit significant between-group differences in most clinical outcomes during the 12-week intervention period. Significant differences were observed in the changes in BMI and visceral-to-subcutaneous fat ratios (Supplementary Table 7). In the low-diversity subgroup, significant between-group differences were observed for changes in body weight (−1.98 ± 0.39 vs. −0.95 ± 0.33 kg; *p* = 0.0483), BMI (*p* = 0.0412), circulating leptin (*p* = 0.0423), and HDL cholesterol (*p* = 0.0342) from the baseline, as summarized in Table 2. Changes in DXA-derived total body fat mass, body fat percentage, and other clinical parameters were not statistically significant in either subgroup. *α*-diversity indices showed divergent temporal patterns between the two subgroups (Figure 3). In the high-diversity subgroup, the diversity declined over time in both groups. No significant between-group differences were observed at week 12 (Figure 3a). In the low-diversity subgroup, significant increases across all *α*-diversity indices were observed in the LP5282-*P* group at week 12 relative to both its own baseline and the placebo group. No temporal changes were detected in the placebo group (Figure 3c). *β*-diversity analysis revealed no significant community-level separation between the treatment groups in the high-diversity subgroup (Figure 3b). In contrast, a significant separation from both the placebo group and its baseline was observed in the LP5282-*P* group at week 12 in the low-diversity subgroup (Figure 3d). Physical activity and sedentary time showed no significant differences among the four subgroups at baseline, week 6, or week 12, for both activity duration and MET-based estimates (Supplementary Tables 8 and 9). Total physical activity summed across weeks 6 and 12, expressed in MET-based values, showed no significant differences among the subgroups (Supplementary Fig. 1). Consistent with this, the three-day dietary records showed no extreme dietary changes across the subgroups. Adjustment for physical activity did not attenuate any of the significant differences involving the LP-L subgroup, and the distinction between PL-L and LP-L was more clearly defined for ΔBMI and Δ*Akkermansia* (Supplementary Table 10).

**Table 2.** Changes in anthropometric, body composition, and metabolic parameters at baseline and week 12 in the low-diversity subgroup.

Parameters	Placebo (Low-diversity, *n* = 26)	LP5282-P (Low-diversity, *n* = 24)	*P*-value
Body weight (kg)			
- visit 2 (baseline)	73.30 ± 1.60	77.44 ± 2.05	0.1144
- visit 4 (after 12 weeks)	72.35 ± 1.72	75.46 ± 2.08	0.2523
- changes from baseline	−0.95 ± 0.33^b^	−1.98 ± 0.39^a^	0.0483
BMI (kg/m^2^)			
- visit 2 (baseline)	26.83 ± 0.25	27.14 ± 0.29	0.4335
- visit 4 (after 12 weeks)	26.52 ± 0.28	26.40 ± 0.34	0.7792
- changes from baseline	−0.31 ± 0.13^b^	−0.74 ± 0.15^a^	0.0412
DXA (fat mass, g)			
- visit 2 (baseline)	25,143.27 ± 777.04	25,468.37 ± 903.33	0.7858
- visit 4 (after 12 weeks)	24,733.36 ± 771.43	24,577.55 ± 880.17	0.8937
- changes from baseline	−410.11 ± 255.13	−890.92 ± 400.24	0.3086
DXA (fat mass, %)			
- visit 2 (baseline)	35.49 ± 1.26	34.57 ± 1.27	0.6117
- visit 4 (after 12 weeks)	35.24 ± 1.26	33.85 ± 1.26	0.4401
- changes from baseline	−0.25 ± 0.28	−0.72 ± 0.42	0.3473
DXA (lean, g)			
- visit 2 (baseline)	46,538.16 ± 1,760.54	48,977.42 ± 1,932.73	0.3544
- visit 4 (after 12 weeks)	46,362.56 ± 1,847.37	48,764.38 ± 1,887.56	0.3678
- changes from baseline	−175.46 ± 260.65	−212.34 ± 271.95	0.9225
Total Cholesterol (mg/dL)			
- visit 2 (baseline)	213.05 ± 8.47	208.74 ± 5.60	0.6769
- visit 4 (after 12 weeks)	205.14 ± 7.24	204.55 ± 5.84	0.9446
- changes from baseline	−7.92 ± 4.60	−4.25 ± 5.36	0.6037
HDL-Cholesterol (mg/dL)			
- visit 2 (baseline)	54.73 ± 2.44	53.92 ± 2.65	0.8217
- visit 4 (after 12 weeks)	54.62 ± 2.36	57.13 ± 2.69	0.4848
- changes from baseline	−0.12 ± 1.21^a^	3.21 ± 0.90^b^	0.0342
LDL-Cholesterol (mg/dL)			
- visit 2 (baseline)	131.14 ± 7.45	131.59 ± 5.04	0.9633
- visit 4 (after 12 weeks)	124.33 ± 5.48	126.42 ± 4.80	0.7749
- changes from baseline	−6.77 ± 5.72	−5.08 ± 4.74	0.8230
Triglycerides (mg/dL)			
- visit 2 (baseline)	127.70 ± 10.12	130.38 ± 13.20	0.8755
- visit 4 (after 12 weeks)	130.25 ± 13.19	118.86 ± 11.94	0.5242
- changes from baseline	2.58 ± 8.97	−11.50 ± 10.07	0.3004
hs-CRP (mg/dL)			
- visit 2 (baseline)	2.97 ± 1.06	5.73 ± 2.70	0.3322
- visit 4 (after 12 weeks)	4.78 ± 2.10	6.81 ± 3.60	0.6219
- changes from baseline	1.81 ± 1.79	1.08 ± 0.99	0.7296
Adiponectin (μg/mL)			
- visit 2 (baseline)	5.00 ± 0.39	5.10 ± 0.46	0.8633
- visit 4 (after 12 weeks)	4.95 ± 0.40	5.41 ± 0.55	0.4947
- changes from baseline	−0.05 ± 0.15	0.30 ± 0.25	0.2224
Leptin (ng/mL)			
- visit 2 (baseline)	11.85 ± 1.38	10.96 ± 1.88	0.7003
- visit 4 (after 12 weeks)	11.61 ± 1.57	8.90 ± 1.56	0.3010
- changes from baseline	−0.24 ± 0.53^b^	−2.07 ± 0.71^a^	0.0423
Visceral fat area (cm^2^)			
- visit 2 (baseline)	124.57 ± 9.97	119.43 ± 7.72	0.6915
- visit 4 (after 12 weeks)	116.94 ± 9.43	105.48 ± 6.69	0.3314
- changes from baseline	−7.60 ± 5.54	−14.02 ± 5.20	0.4045
Subcutaneous fat area (cm^2^)			
- visit 2 (baseline)	246.76 ± 14.19	255.70 ± 16.22	0.6782
- visit 4 (after 12 weeks)	243.85 ± 13.95	238.09 ± 17.81	0.8331
- changes from baseline	−2.95 ± 4.61	−17.61 ± 6.52	0.6975
Total abdominal fat area (cm^2^)			
- visit 2 (baseline)	371.26 ± 14.66	375.11 ± 15.19	0.8554
- visit 4 (after 12 weeks)	360.72 ± 14.79	347.57 ± 16.33	0.7599
- changes from baseline	−10.55 ± 7.93	−27.54 ± 10.51	0.4271
Ratio of visceral/subcutaneous			
- visit 2 (baseline)	0.56 ± 0.06	0.52 ± 0.05	0.6131
- visit 4 (after 12 weeks)	0.53 ± 0.06	0.48 ± 0.04	0.5312
- changes from baseline	−0.03 ± 0.02	−0.04 ± 0.02	0.8447

BMI, body mass index; DXA, dual-energy X-ray absorptiometry; HDL, high-density lipoprotein; LDL, low-density lipoprotein; hs-CRP, high-sensitivity C-reactive protein; Placebo, placebo-treated group; LP5282-P, paraprobiotics derived from *Lactiplantibacillus plantarum* LRCC5282; n, number of participants included in the per-protocol analysis from each low-diversity subgroup. Data are presented as the mean ± SEM. *P*-values represent between-group comparisons, with *P* < 0.05 considered statistically significant. Different superscript letters (a, b) indicate statistically significant differences between groups (*P* < 0.05); groups sharing a letter, or without any superscript, do not differ significantly.

### Taxonomic composition in diversity-stratified subgroups

**Figure 4.**
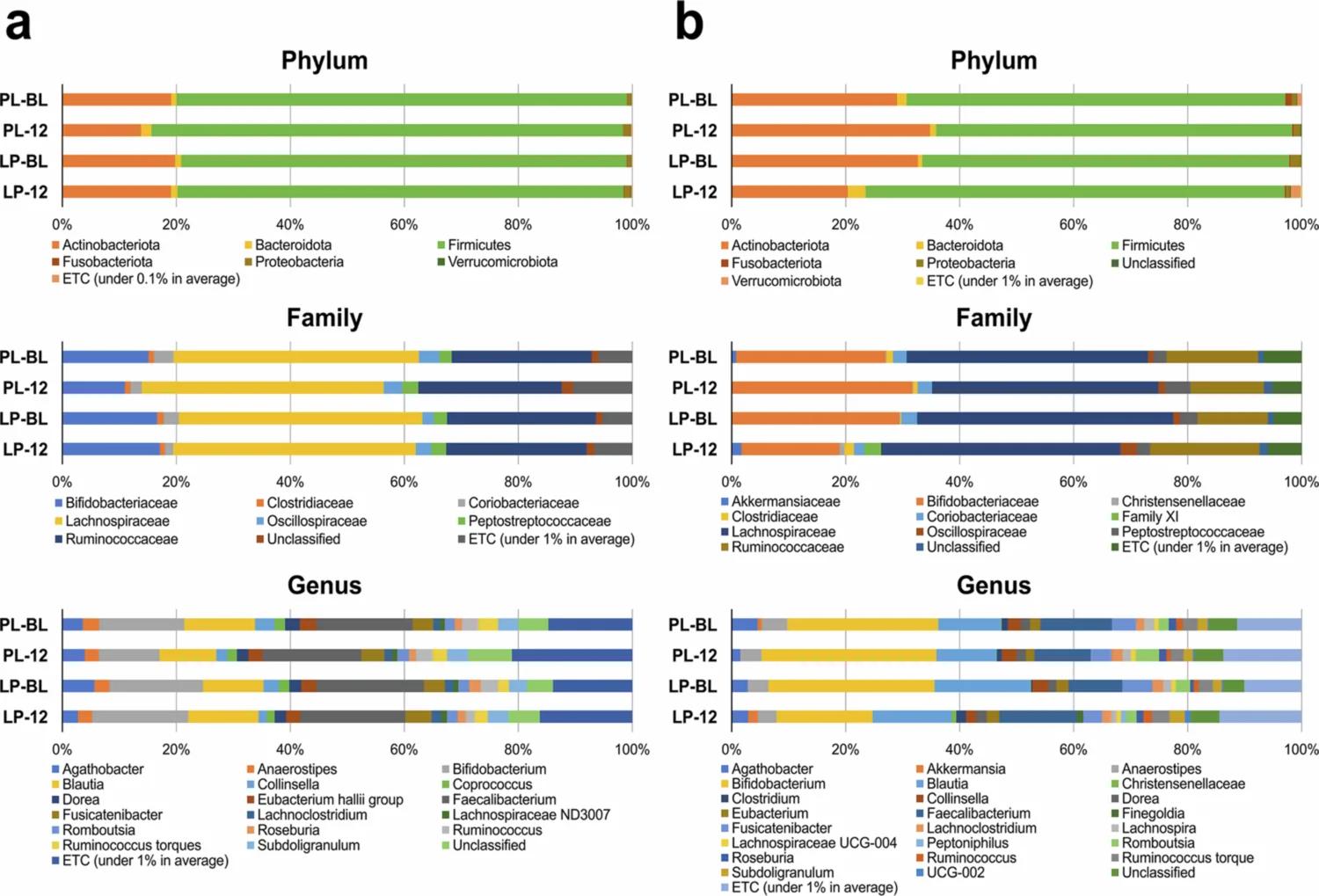
Taxonomic composition of the gut microbiota in diversity-stratified subgroups. (a) Relative abundance of gut microbial taxa at the phylum, family, and genus levels in the high-diversity subgroup. (b) Relative abundance of gut microbial taxa at the phylum, family, and genus levels in the low-diversity subgroup. PL-BL, placebo group at baseline; PL-12, placebo group at week 12; LP-BL, LP5282-*P* group at baseline; LP-12, LP5282-*P* group at week 12. LP5282-*P*, paraprobiotic derived from *Lactiplantibacillus plantarum* LRCC5282. Taxa with a mean relative abundance of less than 1% are grouped as “ETC (under 1% in average)”.

**Figure 5.**
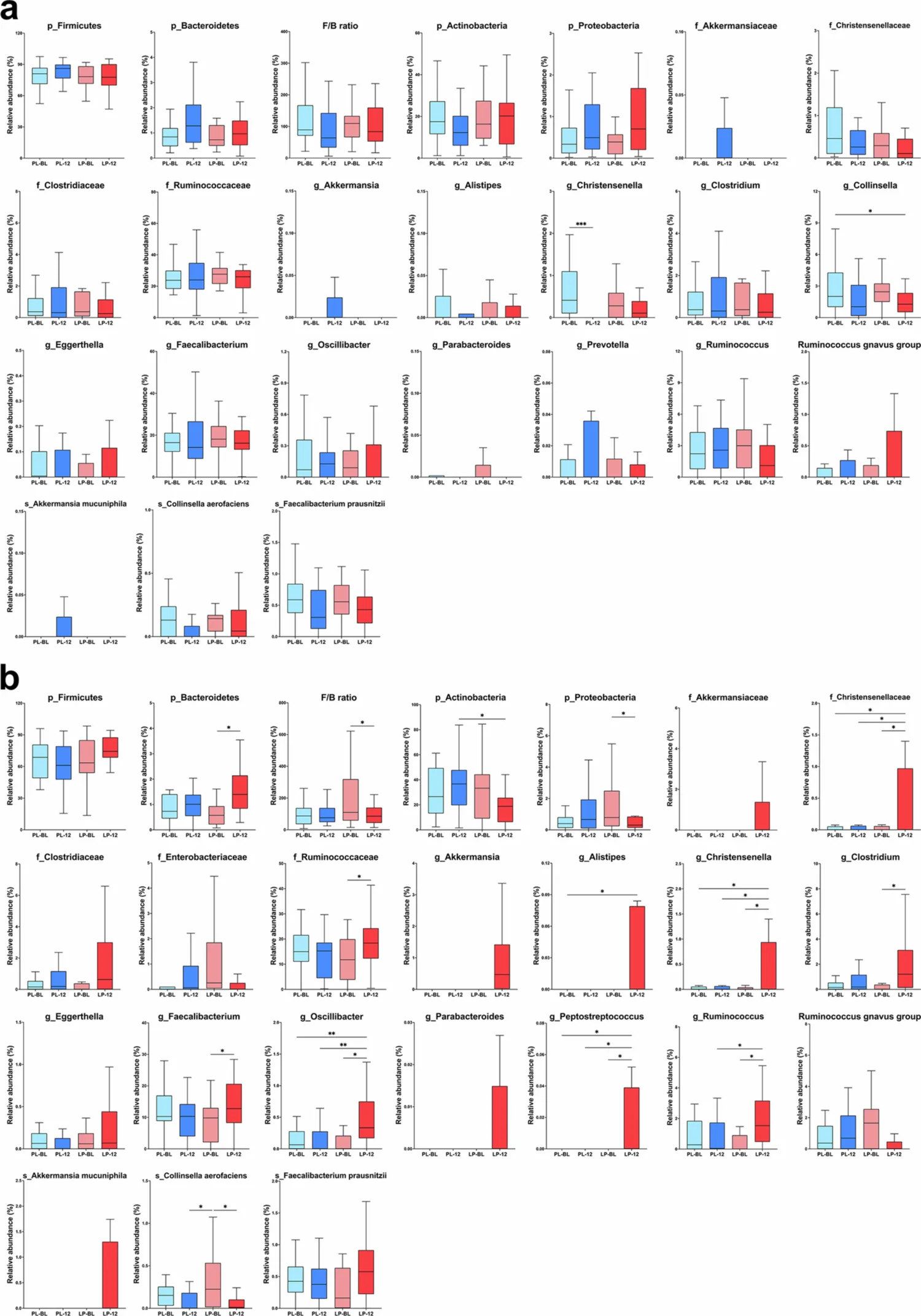
Relative abundance of selected gut microbial taxa in diversity-stratified subgroups. (a) Relative abundance of representative gut microbial taxa at the phylum, family, genus, and species levels in the high-diversity subgroup. (b) Relative abundance of representative gut microbial taxa at the phylum, family, genus, and species levels in the low-diversity subgroup. PL-BL, placebo group at baseline; PL-12, placebo group at week 12; LP-BL, LP5282-*P* group at baseline; LP-12, LP5282-*P* group at week 12. LP5282-*P*, paraprobiotic derived from *Lactiplantibacillus plantarum* LRCC5282. p_, phylum; f_, family; g_, genus; s_, species. F/B ratio, Firmicutes-to-Bacteroidota ratio. Asterisks indicate statistical significance: *p < 0.05; **p < 0.01; ***p < 0.001; ****p < 0.0001.

**Figure 6.**
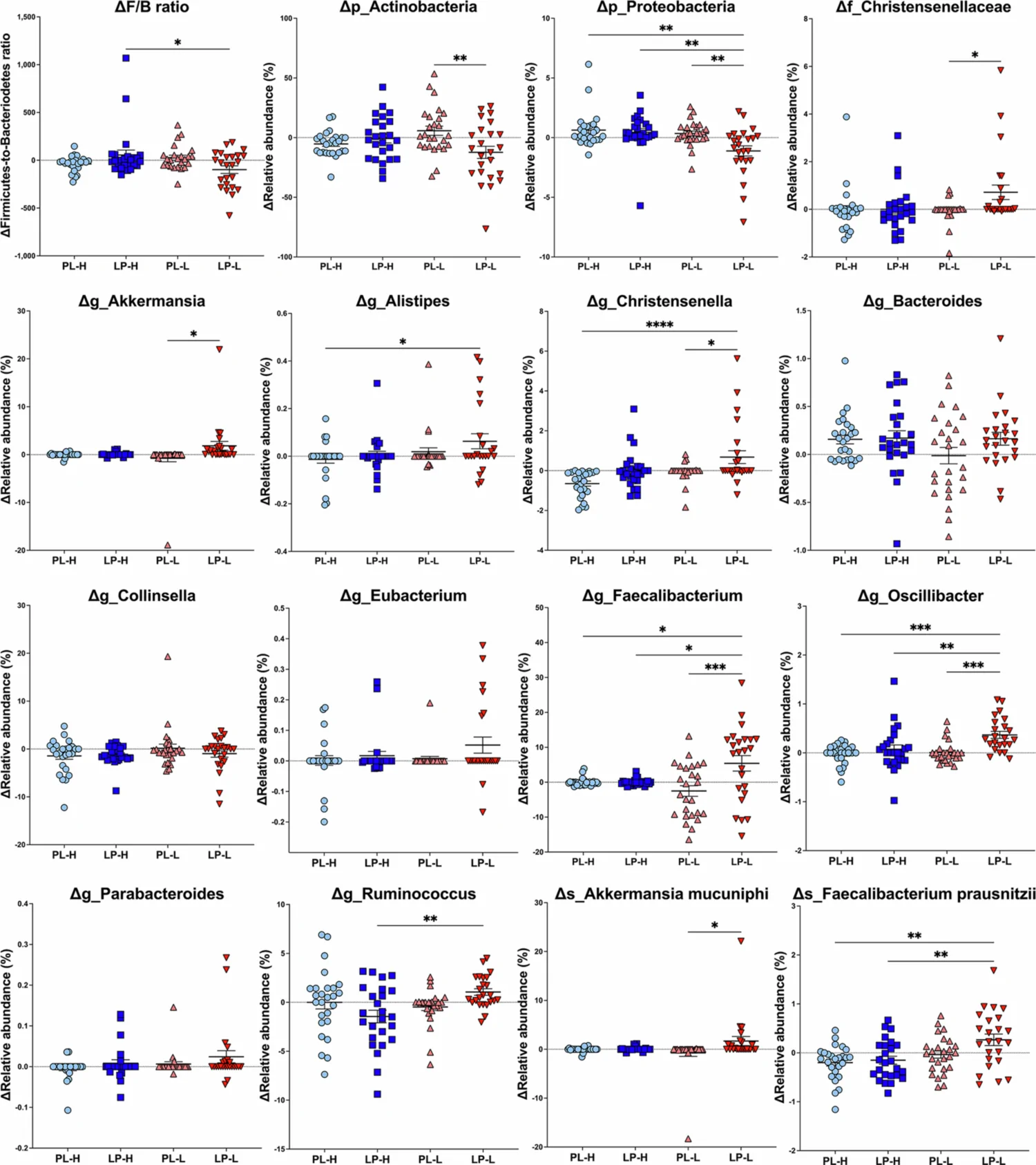
Individual-level changes in gut microbial taxa in diversity-stratified subgroups. PL-H, high-diversity placebo subgroup; LP-H, high-diversity LP5282-*P* subgroup; PL-L, low-diversity placebo subgroup; LP-L, low-diversity LP5282-*P* subgroup; LP5282-*P*, paraprobiotic derived from *Lactiplantibacillus plantarum* LRCC5282; ∆, change from baseline; p_, phylum; f_, family; g_, genus; s_, species. F/B ratio, *Firmicutes*-to-*Bacteroidota* ratio. Change from baseline to week 12 (∆) in the F/B ratio and in the relative abundance of representative gut microbial taxa at the phylum, family, genus, and species levels across all four subgroups. Each point represents an individual participant, and horizontal bars indicate the mean ± SEM within each subgroup. Asterisks indicate statistical significance: *p < 0.05; **p < 0.01; ***p < 0.001; ****p < 0.0001.

The taxonomic composition differed markedly between the two subgroups after LP5282-*P* administration. Phylum-level profiles were broadly conserved at the baseline across all groups (Figure 4). In the high-diversity subgroup, the relative abundances remained stable across all taxonomic ranks, with no significant between-group differences. *Christensenella* showed a significant decrease in the placebo group during the intervention period; however, no intervention-associated compositional shifts were observed (Figure 5a). In contrast, the low-diversity subgroup exhibited a hierarchically consistent pattern of compositional changes after LP5282-*P* administration. The relative abundance of *Bacteroidota* increased significantly compared with that in the placebo group. This was accompanied by a significant reduction in the F/B ratio and concurrent differences in the abundances of *Actinobacteria* and *Proteobacteria* (Figure 5b). These phylum-level shifts were reflected at the family level, and *Christensenellaceae* and *Ruminococcaceae* were significantly enriched. This pattern extended to the genus level, where *Christensenella*, *Alistipes*, *Oscillibacter*, *Faecalibacterium*, *Ruminococcus*, and *Peptostreptococcus* showed significant differences between the baseline and placebo groups. In contrast, *Clostridium* exhibited an increased relative abundance relative to its baseline within the LP5282-*P* group. At the species level, the relative abundance of *Collinsella aerofaciens* showed a significant between-group difference. In contrast, *A. muciniphila* and *F. prausnitzii* showed no significant differences between groups. Sequences assigned to the genus *Lactiplantibacillus* remained negligible across all groups at both baseline and week 12, and no ASVs were resolved to *L. plantarum* at the species level in any fecal sample. The corresponding individual-level changes from baseline to week 12 are shown in Figure 6. Significant differences were restricted to comparisons involving LP-L, in which the directional changes in the F/B ratio, *Christensenellaceae, Christensenella, Alistipes*, *Faecalibacterium, Oscillibacter, Akkermansia*, and *F. prausnitzii* were concordant with the group-level differences described above. In contrast, *Bacteroides, Collinsella, Eubacterium*, and *Parabacteroides* showed no significant between-group differences.

### Differential abundance and functional inference in the diversity-stratified subgroups

**Figure 7.**
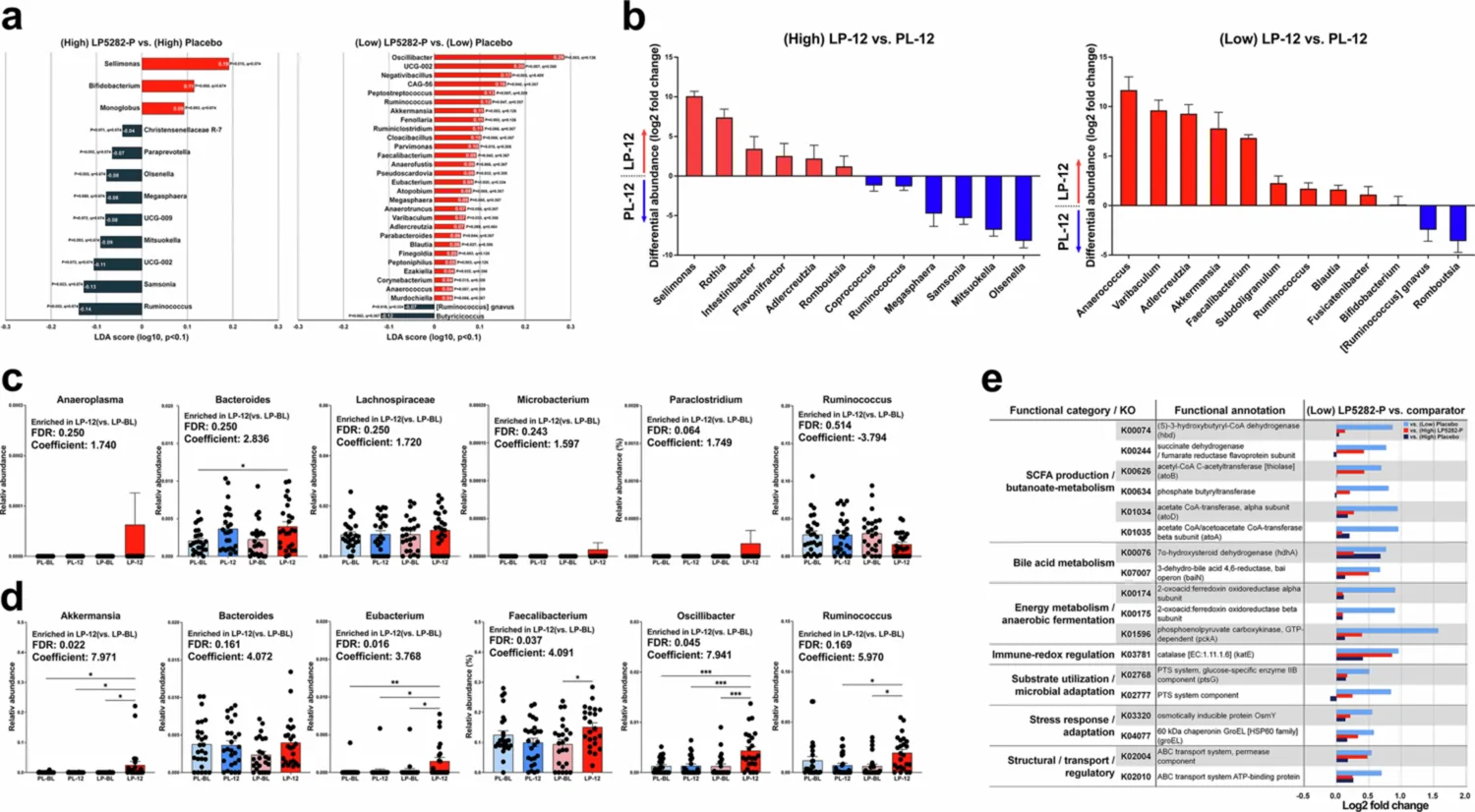
Differential abundance analysis and predicted functional profiles in diversity-stratified subgroups. (a) Differentially abundant taxa identified by LEfSe in the high-diversity (left) and low-diversity (right) subgroups, expressed as LDA scores (log10; *p* < 0.1). (b) Differential abundance by ANCOM-BC in the high-diversity (left) and low-diversity (right) subgroups, expressed as log2 fold change of LP-12 relative to PL-12. (c) Multivariable associations between microbial taxa and LP5282-*P* administration identified by MaAsLin2 in the high-diversity subgroup. (d) Multivariable associations between microbial taxa and LP5282-*P* administration identified by MaAsLin2 in the low-diversity subgroup. (e) Predicted functional profiles inferred by PICRUSt2 in LP-L relative to PL-L, LP-H, and PL-H, expressed as log2 fold change and annotated against KEGG orthologs by functional category. PL-BL, placebo group at baseline; PL-12, placebo group at week 12; LP-BL, LP5282-*P* group at baseline; LP-12, LP5282-*P* group at week 12. LP5282-*P*, paraprobiotic derived from *Lactiplantibacillus plantarum* LRCC5282. LP-H, high-diversity LP5282-*P* subgroup; LP-L, low-diversity LP5282-*P* subgroup; PL-H, high-diversity placebo subgroup; PL-L, low-diversity placebo subgroup. LEfSe, linear discriminant analysis effect size; ANCOM-BC, analysis of composition of microbiomes with bias correction; MaAsLin2, multivariable association with linear models 2; PICRUSt2, phylogenetic investigation of communities by reconstruction of unobserved states 2; KO, KEGG ortholog; FDR, false discovery rate. Asterisks indicate statistical significance: *p < 0.05; **p < 0.01; ***p < 0.001; ****p < 0.0001.

The LEfSe, ANCOM-BC, and MaAsLin2 analyses revealed divergent compositional signals between the two subgroups (Figure 7). In the high-diversity subgroup, LEfSe (Figure 7a) and ANCOM-BC (Figure 7b) identified taxa that were differentially abundant between groups, including the enrichment of *Sellimonas* and related taxa in LP-H and *Ruminococcus* and *Megasphaera* in PL-H. However, MaAsLin2 yielded no statistically significant taxon-level associations after false discovery rate correction (Figure 7c), indicating the absence of robust intervention-associated compositional signals in this subgroup. In the low-diversity subgroup, consistent signals emerged across all the three analytical frameworks. LEfSe identified multiple taxa that were enriched in LP-L, including *Oscillibacter*, *Akkermansia*, *Ruminococcus*, *Faecalibacterium*, and *Peptostreptococcus*. *Ruminococcus gnavus* and *Butyricoccus* were abundant in the PL-L group (Figure 7a). ANCOM-BC corroborated the enrichment of *Akkermansia*, *Faecalibacterium*, *Subdoligranulum*, *Ruminococcus*, and *Blautia* in LP-L compared to PL-L. In contrast, *Fusicatenibacter*, *Bifidobacterium*, *Ruminococcus gnavus*, and *Romboutsia* were less abundant in the LP-L group (Figure 7b). MaAsLin2 adjusted for age, sex, and baseline BMI confirmed a significant positive association with *Akkermansia*, *Eubacterium*, *Faecalibacterium*, and *Oscillibacter*. These associations were independent of the baseline host characteristics (Figure 7d).

The predicted functional capacity was inferred using PICRUSt2 and annotated against the KEGG orthologs (KOs), with LP-L used as the reference group for PL-L, LP-H, and PL-H (Figure 7e). LP-L showed a higher predicted abundance of KOs annotated to SCFA production, butanoate metabolism, bile acid metabolism, and anaerobic energy fermentation than the other three comparators. The most pronounced difference was observed in the PL-L group. KOs annotated to immune redox regulation, substrate utilization, stress response, and structural and transport functions also showed a higher predicted abundance in LP-L than in PL-L. Differences relative to the LP-H and PL-H levels were also attenuated. Relative to PL-H, a few KOs in the SCFA production and substrate utilization categories approached zero, whereas most remained positive. No consistent directionality was observed among the remaining comparators.

### Fecal metabolite profiles in diversity-stratified subgroups

**Figure 8.**
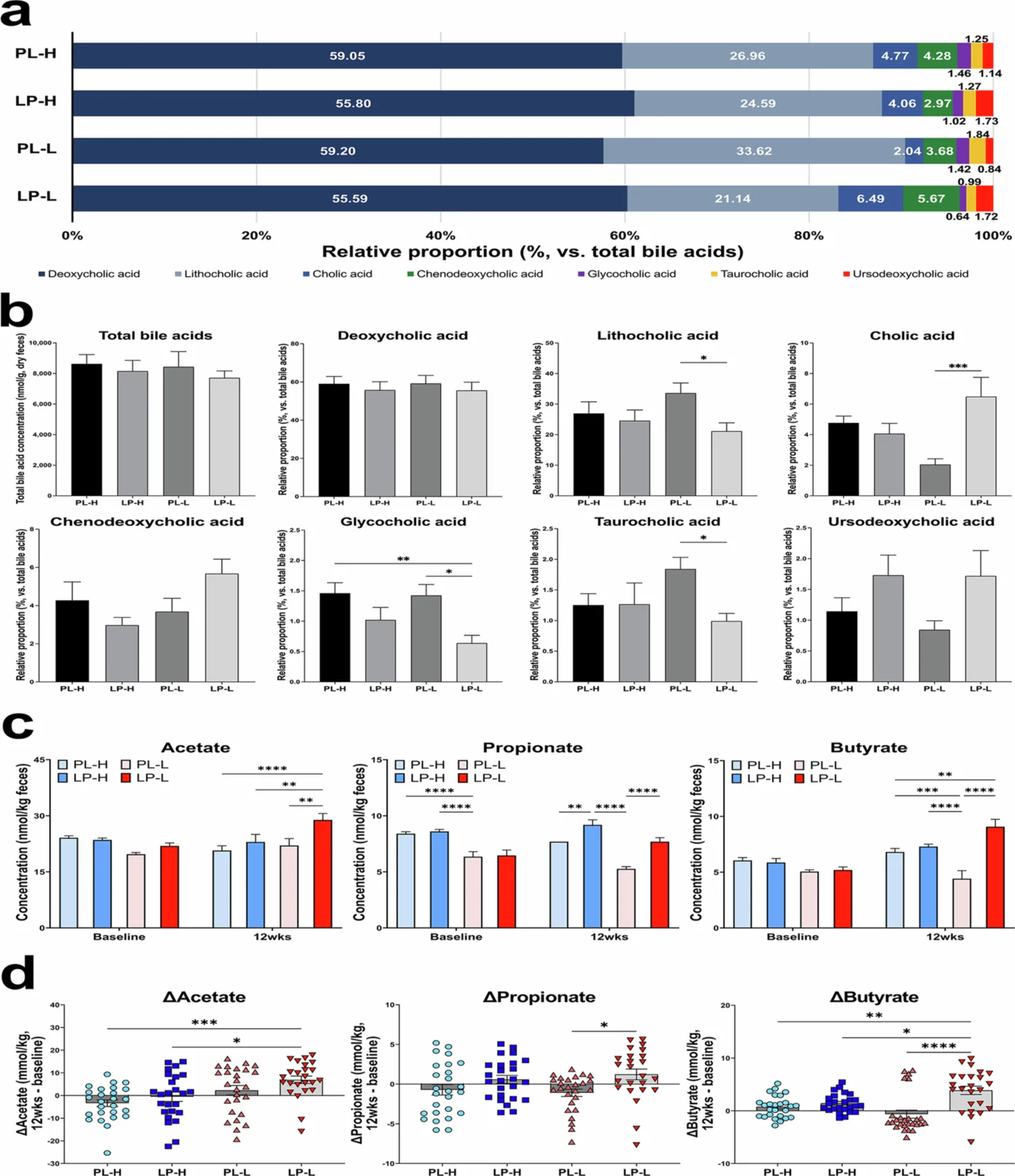
Fecal bile acid and short-chain fatty acid profiles in diversity-stratified subgroups. (a) Relative proportions of seven fecal bile acid species as a percentage of total bile acids at week 12 across all four subgroups. (b) Total fecal bile acid concentration and relative proportions of individual bile acid species at week 12 across all four subgroups. (c) Fecal concentrations of acetate, propionate, and butyrate at baseline and week 12 across all four subgroups. (d) Individual-level changes in fecal acetate, propionate, and butyrate. LP-H, high-diversity LP5282-*P* subgroup; LP-L, low-diversity LP5282-*P* subgroup; PL-H, high-diversity placebo subgroup; PL-L, low-diversity placebo subgroup. LP5282-*P*, paraprobiotic derived from *Lactiplantibacillus plantarum* LRCC5282. Data in (b) are presented as mean ± SEM. Asterisks indicate statistical significance: *p < 0.05; **p < 0.01; ***p < 0.001; ****p < 0.0001.

Fecal bile acid and SCFA concentrations were assessed across all four subgroups at baseline and at week 12 (Figure 8). Total fecal bile acid concentrations were comparable across subgroups (PL-H: 8,628.43 ± 604.42; LP-H: 8,439.27 ± 992.96; PL-L: 8,154.21 ± 705.56; LP-L: 7,827.37 ± 488.54 nmol/g dry feces). Deoxycholic acid was the predominant species in all groups. Despite this overall similarity, the bile acid composition profiles differed significantly in the low-diversity subgroup. Supplementation was associated with a significant increase in the proportion of cholic acid and a concurrent reduction in lithocholic acid, glycocholic acid, and taurocholic acid levels relative to the placebo. No corresponding differences were detected in the high-diversity subgroups (Figure 8a, b). SCFA concentrations were comparable across all subgroups at baseline (Figure 8c). At week 12, acetate and butyrate levels were significantly elevated in the LP-L group compared to those in both placebo subgroups. No corresponding differences were observed in the LP-H group. Propionate levels were significantly higher in the LP-H and LP-L groups than in the PL-L group; however, they were significantly lower in the LP-L group than in the LP-H group. Individual-level changes in fecal SCFA concentrations from baseline to week 12 are shown in Figure 8d. In the LP-L group, butyrate increased significantly relative to all other subgroups, and acetate relative to both high-diversity subgroups. The significant increase in propionate was confined to the comparison with the low-diversity placebo group.

### Differential clinical responses across diversity subgroups

The treatment-by-diversity subgroup interaction analysis revealed divergent patterns of clinical change across the four subgroups over the 12-week intervention period (Table 3). Changes in the body weight and BMI from baseline differed significantly across subgroups. Specifically, significant reductions were observed in the LP-L group but not in the LP-H group. The placebo subgroup showed no significant differences. Significant reductions in leptin levels and subcutaneous fat area were similarly confined to the LP-L group relative to all other subgroups. HDL cholesterol levels showed a contrasting directional pattern: a significant increase was observed in the LP-L group, whereas a non-significant decrease was observed in the LP-H group. In the interaction analysis, the changes from baseline in DXA-derived fat mass, total cholesterol, LDL cholesterol, triglyceride, adiponectin, visceral fat area, and total abdominal fat area did not reach statistical significance.

**Table 3.** Clinical and metabolic responsiveness to paraprobiotic intervention stratified by baseline gut microbial diversity.

Parameter	High-diversity subgroup		Low-diversity subgroup	
	Placebo (*n* = 26)	LP5282-P (*n* = 25)	Placebo (*n* = 26)	LP5282-P (*n* = 24)
∆BW (kg)	−1.25 ± 0.36^ab^	−0.60 ± 0.23^b^	−0.95 ± 0.33^ab^	−1.98 ± 0.39^a^
∆BMI (kg/m^2^)	−0.54 ± 0.12^ab^	−0.22 ± 0.07^b^	−0.31 ± 0.13^ab^	−0.74 ± 0.15^a^
∆DXA (mass, g)	−730.24 ± 313.23	−278.14 ± 235.50	−410.11 ± 255.13	−890.92 ± 400.24
∆TC (mg/dL)	−8.36 ± 4.80	0.76 ± 6.52	−7.92 ± 4.60	−4.25 ± 5.36
∆HDL-C (mg/dL)	0.69 ± 1.09^ab^	−1.16 ± 1.33^a^	−0.12 ± 1.21^ab^	3.21 ± 0.90^b^
∆LDL-C (mg/dL)	−4.27 ± 4.49	−2.32 ± 8.07	−6.77 ± 5.72	−5.08 ± 4.74
∆TG (mg/dL)	3.96 ± 13.31	2.96 ± 9.46	2.58 ± 8.97	−11.50 ± 10.07
∆Adiponectin (μg/mL)	−0.31 ± 0.29	−0.19 ± 0.25	−0.05 ± 0.15	0.30 ± 0.25
∆Leptin (ng/mL)	−1.27 ± 1.19^b^	−0.71 ± 0.87^b^	−0.24 ± 0.53^b^	−2.07 ± 0.71^a^
∆VFA (cm^2^)	−12.60 ± 5.02	−1.61 ± 6.16	−7.60 ± 5.54	−14.02 ± 5.20
∆SFA (cm^2^)	−4.52 ± 5.48^b^	−7.22 ± 9.62^b^	−2.95 ± 4.61^b^	−17.61 ± 6.52^a^
∆TFA (cm^2^)	−17.12 ± 8.91	−13.11 ± 10.03	−10.55 ± 7.93	−27.54 ± 10.51

∆, change from baseline (week 12 minus baseline); BW, body weight; BMI, body mass index; DXA, dual-energy X-ray absorptiometry; TC, total cholesterol; HDL-C, high-density lipoprotein cholesterol; LDL-C, low-density lipoprotein cholesterol; TG, triglycerides; VFA, visceral fat area; SFA, subcutaneous fat area; TFA, total abdominal fat area; LP5282-P, paraprobiotic derived from Lactiplantibacillus plantarum LRCC5282; n, number of participants included in the per-protocol analysis from each treatment group within each diversity subgroup. Data are presented as mean ± SEM. Different superscript letters (a, b) indicate statistically significant differences among the four subgroups (*P* < 0.05); subgroups sharing a letter, or without any superscript, do not differ significantly.

### Microbial, metabolite, and clinical outcome correlations in the low-diversity subgroup

**Figure 9.**
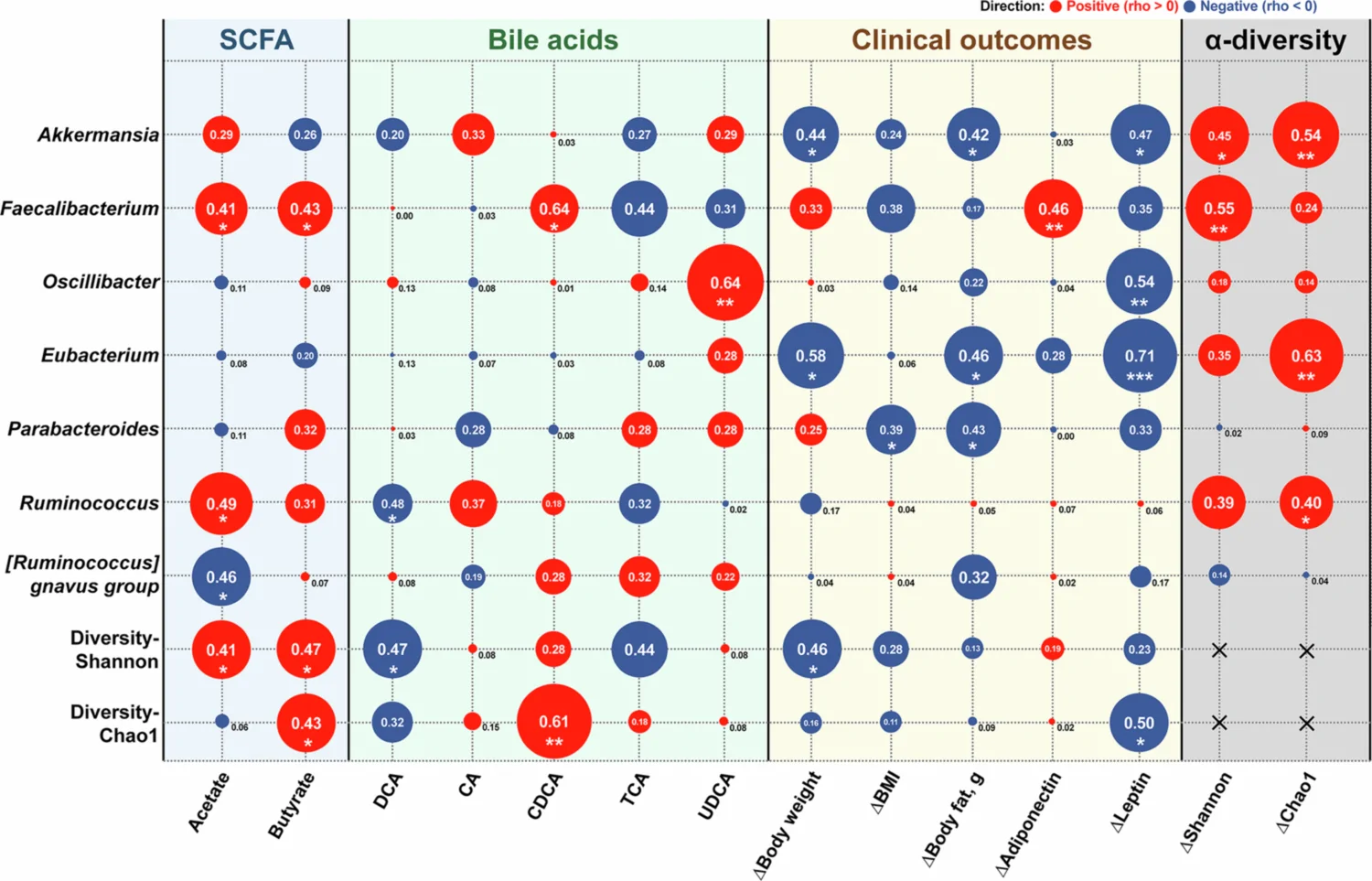
Spearman correlations of gut microbial taxa and *α*-diversity indices with fecal metabolites and clinical outcomes in the low-diversity subgroup. Bubble plot depicting Spearman correlation coefficients (rho) of changes in the relative abundance of representative gut microbial taxa and in the Shannon and Chao1 *α*-diversity indices with fecal short-chain fatty acid concentrations, fecal bile acid proportions, and changes in clinical outcome parameters within the low-diversity subgroup. Bubble color indicates the direction of correlation: red, positive (rho > 0); blue, negative (rho < 0). Bubble size is proportional to the absolute value of the correlation coefficient. Numerical values within bubbles represent Spearman *rho* values. SCFA, short-chain fatty acid; DCA, deoxycholic acid; CA, cholic acid; CDCA, chenodeoxycholic acid; TCA, taurocholic acid; UDCA, ursodeoxycholic acid; BMI, body mass index; ∆, change from baseline. Asterisks indicate statistical significance: *p < 0.05; **p < 0.01; ***p < 0.001.

Spearman correlation analyses within the low-diversity subgroup revealed associations between changes in relative microbial abundance, fecal metabolite concentrations, and clinical outcome parameters (Figure 9). The taxa examined comprised those identified as differentially abundant or associated with LP5282-*P* across the LEfSe, ANCOM-BC, and MaAsLin2 analyses. This set was supplemented by genera with established roles in short-chain fatty acid or bile acid metabolism and by recognized markers of dysbiosis, thereby encompassing both beneficial and potentially detrimental taxa. The Shannon and Chao1 *α*-diversity indices were also included to examine their correlations with fecal metabolites and clinical parameters. *Faecalibacterium* and *Ruminococcus* were significantly and positively correlated with fecal acetate content. *Faecalibacterium* was also correlated with butyrate and *R. gnavus* was positively associated with acetate. *Faecalibacterium* abundance was positively correlated with CDCA and TCA. *Oscillibacter* was significantly positively correlated with UDCA, whereas *Ruminococcus* was negatively correlated with DCA. Changes in the abundances of *Akkermansia* and *Eubacterium* were significantly negatively correlated with changes in body weight, body fat mass, and leptin. Increases in the abundance of the following taxa also corresponded to reductions in adiposity-related parameters: *Oscillibacter* abundance was significantly negatively correlated with changes in leptin levels, *Faecalibacterium* was positively correlated with changes in adiponectin levels, and *Parabacteroides* was negatively correlated with changes in both BMI and body fat mass. No significant associations were detected between the remaining taxa and the clinical outcome measures. The *α*-diversity indices showed correlations with the same metabolites and clinical parameters as the responsive taxa. The Shannon index correlated positively with fecal acetate and butyrate, and the Chao1 index with butyrate. Among the bile acids, the Shannon index correlated negatively with DCA, and the Chao1 index positively with CDCA. For the clinical parameters, the Shannon index correlated negatively with changes in body weight, and the Chao1 index with changes in leptin.

## Discussion

Microbiota-targeted interventions have been extensively investigated as potential strategies for managing obesity. However, randomized controlled trials have consistently yielded heterogeneous and clinically modest outcomes at the population level,^10^ implicating the pre-existing characteristics of the resident gut microbiota as a potential determinant of individual responsiveness.^16^ ^,^ ^17^ The present study evaluated the clinical, microbiome, and fecal metabolite responses to LP5282-*P*, a paraprobiotic derived from *L. plantarum* LRCC5282,^21^ in a 12-week multicenter, randomized controlled trial in overweight adults. Stratified analyses based on baseline gut microbial *α*-diversity were applied to determine the extent to which pre-existing microbial community characteristics were associated with variability in paraprobiotic responsiveness across clinical, compositional, and metabolite outcome domains.

In the overall PP population, body weight, BMI, and DXA-derived total body fat mass decreased over the 12-week intervention period in both groups, with no statistically significant between-group differences observed in any clinical outcome. Several factors may have constrained the detection of treatment effects at the population level. Discordance between pre-specified endpoints and the biological pathways through which microbiota-targeted interventions exert their effects, together with the absence of stratification for baseline microbiome heterogeneity and the limited duration of the intervention, have been identified as recurrent determinants of attenuated effect sizes in this field.^48^ DXA-derived total body fat mass has been reported to exhibit limited responsiveness to short-term microbiota-targeted interventions compared to anthropometric indices.^8^ ^,^ ^24^ The broad distribution of baseline gut microbial diversity across the study population may have further reduced the statistical resolution of population-averaged treatment comparisons.^16^ ^,^ ^17^ Hence, prospective trials incorporating extended intervention periods and more refined eligibility criteria are warranted.^48^ These study designs will help to establish the conditions under which LP5282-*P* confers adiposity-related benefits across a phenotypically heterogeneous population of overweight individuals.

The relationship between baseline gut microbial *α*-diversity and responsiveness to microbiome-targeted interventions has been examined in several prior studies, although findings have been inconsistent in both direction and outcome domain. Alcázar et al.^33^ reported that children with obesity above a defined Simpson index threshold exhibited greater improvements in metabolic risk scores, HDL cholesterol levels, and systolic blood pressure after a structured lifestyle intervention, suggesting that higher baseline diversity may favor clinical responsiveness. Regarding microbiome compositional responses, Klimenko et al.^17^ reported that baseline *α*-diversity was positively associated with the magnitude of *β*-diversity change across eight distinct dietary interventions, suggesting that higher-diversity communities may retain higher adaptive capacity under perturbation. In contrast, Sardá et al.^49^ demonstrated that significant community-level restructuring occurred exclusively in participants with a *Prevotella*-rich baseline microbiota cluster, whereas those with a *Bacteroides*-rich cluster showed no corresponding shift, indicating that the compositional context may hinder microbiome responsiveness independently of diversity. Notably, the relationship between baseline diversity and responsiveness was not uniform, even within a single study. Wastyk et al.^18^ observed that immunological response trajectories to a high-fiber diet diverged according to baseline microbiota diversity. Participants with lower microbial diversity showed an increased trajectory rather than an attenuated inflammation. In contrast, a fermented food intervention in the same cohort was associated with diversity gains and reductions in inflammation, which were more pronounced in lower-diversity participants. This pattern illustrates that the direction of the diversity-responsiveness relationship is conditioned by the specific nature of the intervention rather than by baseline diversity alone.^18^ In the present study, LP5282-*P*-associated responses were observed exclusively in the low-diversity subgroup, encompassing reductions in body weight, BMI, and leptin, an increase in HDL cholesterol, and concurrent increases in *α*-diversity and *β*-diversity separation. The high-diversity subgroup showed only isolated differences in BMI and the visceral-to-subcutaneous fat ratio, without coherent multi-domain changes. The convergence of the clinical and microbiome-level responses within the same subgroup suggests that these two axes of responsiveness are not independent. Instead, they likely reflect shared susceptibility conditioned by pre-existing microbial characteristics.^16^ The underlying basis of this susceptibility warrants further investigation.

The structural properties of low baseline *α*-diversity may provide a conceptual framework for interpreting this pattern. *α*-diversity encompasses both the richness and evenness of taxa within a community, reflecting the internal structural complexity of the gut microbiota. *β*-diversity captures compositional dissimilarity between communities. The concurrent changes in both *α*- and *β*-diversity in the low-diversity subgroup are consistent with LP5282-*P* contributing to shifts in individual taxa and in overall community composition. Low gut microbial gene richness has been associated with pronounced adiposity, insulin resistance, dyslipidemia, and systemic inflammation in large-scale metagenomic cohort studies.^7^ The low-diversity dysbiotic state is characterized as one wherein the complex anaerobic community of a healthy adult gut is replaced by a functionally impaired community with a reduced diversity.^15^ Therefore, the low-diversity state observed at baseline in the LP-L group does not reflect a quantitative difference in species number alone; it represents a qualitatively distinct condition characterized by functional community impairment and reduced structural stability. Species-rich communities are less susceptible to compositional reorganization because the constituent taxa use limited resources more efficiently and collectively occupy a denser network of nutritional niches.^11^ This principle has been experimentally validated in the context of the gut microbiota, where colonization resistance has been shown to increase substantially with community diversity through the competitive consumption of shared nutritional resources.^16^ In contrast, communities with low diversity harbor a less densely occupied niche. This condition permits broader and more coherent compositional reorganization in response to external stimuli. Direct experimental support for this hypothesis comes from studies demonstrating that the initial state of the gut microbiome determines the nature and magnitude of its responses to external perturbations. For instance, Raymond et al.^50^ showed that gut microbiome restructuring following antibiotic exposure is determined by the baseline microbiome state, with lower baseline diversity associated with more pronounced community-level changes. Zmora et al.^19^ further reported that mucosal colonization responses to probiotic administration depend on baseline host and microbiome features, with the resident microbiome composition as the principal determinant of responsiveness to exogenous stimuli. In LP-L, this selective response may reflect the lower niche occupancy and functional redundancy of low-diversity communities, which render them more susceptible to compositional change. In contrast, high-diversity communities, with greater niche occupancy and redundancy, may be more resistant to comparable perturbation. The concurrent *α*- and *β*-diversity changes in the LP-L group, and the absence of corresponding changes in the high-diversity subgroup, are consistent with, although not sufficient to establish, this interpretation. The decline in *α*-diversity observed across both arms of the high-diversity subgroup is more parsimoniously attributed to temporal fluctuation around individual equilibrium values. This pattern is consistent with longitudinal microbiome dynamics, wherein diversity indices exhibit substantial intra-individual variation while tending to return to individual-specific baseline levels.^51^ Although the relationship between baseline diversity and intervention responsiveness is not consistent across studies, with both its direction and magnitude varying by intervention type,^18^ the present findings indicate that responses associated with LP5282-*P* were more pronounced in participants with low baseline diversity. As noted above, this state provides conditions that permit community-level reorganization. Beyond this diversity-conditioned pattern, the non-viable formulation of LP5282-*P* constrains causal attribution of these changes to the intervention. Because LP5282-*P* consists of non-viable cells, it is not expected to replicate or stably colonize the gut. The absence of a detectable species-level *L. plantarum* signal therefore neither confirms nor excludes biological activity, because amplicon sequencing of a non-viable preparation assesses neither cell viability nor direct molecular action. Nevertheless, the compositional, predicted-functional, and metabolite-level changes were confined to the low-diversity LP5282-*P* subgroup, with no counterpart in the corresponding placebo subgroup, and the taxonomic associations persisted after adjustment for age, sex, and baseline BMI. Previous human studies of non-viable *L. plantarum* and related postbiotic preparations have supported mechanistic interpretation by relating administration to complementary intermediate readouts, including changes in the resident gut microbiota, fecal metabolite profiles, inferred functional pathways, and downstream host responses.^52-54^ The present study showed a broadly comparable cross-domain pattern, in which microbial compositional shifts, altered fecal short-chain fatty acid and bile acid profiles, PICRUSt2-inferred functional changes, and physiological outcomes occurred predominantly in the low-diversity subgroup. However, because strain-specific tracking, strain-associated metabolites, and proximal host-response markers were not assessed, the initial biological connection between LP5282-*P* administration and the subsequent microbial and clinical responses could not be established. The observed cross-domain coherence therefore provides indirect support for the possibility that LP5282-*P* contributed to reshaping the resident microbial community and its associated metabolic responses, rather than evidence of a strain-specific causal mechanism. Accordingly, the enriched taxa should be interpreted as components of an LP5282-*P*-associated restructuring of the resident microbial community, rather than as direct indicators of LRCC5282 exposure or strain-specific biological activity.

Shifts in obesity-associated taxa may be part of the microbial response that connects microbiota-targeted interventions with host metabolic outcomes. *Proteobacteria* enrichment is a hallmark of gut dysbiosis. The expansion of this phylum has been linked to increased LPS production, metabolic endotoxemia, and systemic inflammation in obesity, whereas its reduction has been associated with improvements in gut barrier integrity and metabolic parameters.^1^ The F/B ratio operates within a similar metabolic framework. Elevated F/B ratios have been linked to increased energy-harvesting capacity and adiposity-related dysfunction, whereas lower ratios have been proposed as indicators of a more favorable microbial composition.^2^ ^,^ ^3^ Among the taxa associated with metabolically healthy obesity phenotypes, *Christensenellaceae* and *Christensenella* are less abundant in metabolically unhealthy obese individuals. Positive associations with HDL cholesterol and inverse associations with triglycerides and visceral adiposity have also been consistently reported, suggesting a role in lipid metabolism regulation.^55^ ^,^ ^56^ *Faecalibacterium*, the principal butyrate-producing genus, is frequently depleted in obesity-associated dysbiosis. Inverse correlations between its abundance and the markers of systemic inflammation and adiposity support its role as an indicator of gut microbial health.^57^ Similarly, *Oscillibacter* was negatively associated with metabolically unhealthy obesity in another large-scale cohort study.^58^ *Alistipes* has been proposed to contribute to gut immune homeostasis, with a reduced abundance commonly observed under inflammatory conditions.^59^ *A. muciniphila* has emerged as a well-characterized microbiome-associated taxon in the context of metabolic health. Consistent inverse associations have been reported between its abundance and body fat mass, insulin resistance, and obesity-related metabolic parameters in multiple human cohorts. A higher baseline abundance of this taxon has also been associated with more favorable metabolic responses during dietary interventions.^25^

Several community-level shifts observed in LP-L can be compared with findings from previous *L. plantarum* intervention studies, including those using non-viable preparations. In our earlier preclinical study, LP5282-*P* attenuated obesity-associated dysbiosis in high-fat-diet models, offering a relevant comparison for the present LP-L profile.^21^ At the community level, lower *Firmicutes*-to-*Bacteroidota* ratios have been reported following administration of heat-killed *L. plantarum* L-137 and heat-inactivated FRT4 or LP5282-*P*, with viable *L. plantarum* Dad-13 eliciting a comparable shift, namely a decrease in *Firmicutes* accompanied by an increase in *Bacteroidota*.^21^ ^,^ ^53^ ^,^ ^60^ ^,^ ^61^ At finer taxonomic resolution, *Bacteroides* was enriched following administration of L-137, FRT4, and LP5282-*P*, and *Alistipes* following heat-inactivated FRT4 or LP5282-*P* in obesity models.^21^ ^,^ ^53^ ^,^ ^61^ Heat-inactivated FRT4 further increased *Alloprevotella, Lactobacillus*, and *Muribaculaceae*-related taxa and reduced *Romboutsia, Odoribacter, Blautia, Bilophila*, and *Anaerotruncus*.^61^ In the same model, LP5282-*P* increased *Dorea*, *Macellibacteroides, Desulfovibrio*, and *Bacteroides* across both the alleviation and prophylactic models, with further enrichment of *Alistipes* and the family *Oscillospiraceae* in the prophylactic model.^21^ In human studies, viable Dad-13 was associated with trends toward increased *Prevotella* and reduced *Collinsella* abundances.^60^ A multi-strain fermented postbiotic containing *L. plantarum* *p*-8 enriched several *Faecalibacterium prausnitzii* lineages, raised fecal butyrate concentrations, and reduced *Ruminococcus gnavus* and *Megamonas funiformis*, although the contributions of individual strains could not be resolved.^54^ These findings partially overlap with the LP-L profile, particularly the enrichment of *Alistipes* and *Faecalibacterium,* the increased abundance of *Oscillospiraceae*-related taxa, and the lower abundances of *R. gnavus, Romboutsia*, and *Collinsella aerofaciens*. Some taxa, however, did not align with previous reports. The direction and magnitude of change differed for several taxa, including *Bacteroides*, which was consistently enriched in the preclinical and heat-killed preparation studies but showed no significant difference in LP-L. These differences may be attributable to variation in host species, preparation, and dose across studies. The overall concordance in taxonomic and metabolite-level responses across previous *L. plantarum* interventions supports the biological plausibility of the LP5282-*P*-associated compositional changes in the resident gut microbiota observed in the present study.

Microbiome compositional data are inherently subject to compositional bias and sensitivity to normalization strategies. Cross-method concordance across complementary analytical frameworks has been proposed as a criterion for evaluating the robustness of differential abundance findings.^62^ In the high-diversity subgroup, the absence of significant taxon-level associations under MaAsLin2, despite the signals identified by LEfSe and ANCOM-BC, indicated that the observed between-group differences were not independent of the baseline host characteristics. Therefore, these findings are unlikely to reflect robust intervention-associated compositional signals. In the low-diversity subgroup, the convergence of consistent signals across all three frameworks provided methodologically robust support for the biological relevance of these associations. The associations of *Akkermansia, Eubacterium, Faecalibacterium,* and *Oscillibacter* with LP5282-*P* administration were confirmed using MaAsLin2 after adjusting for age, sex, and baseline BMI. Notably, *Faecalibacterium* and *Oscillibacter* were also among those enriched in the relative abundance analyses. This consistency indicates that their association with LP5282-*P* administration in the low-diversity context is unlikely to be an artifact of any single analytical approach, but rather reflects a compositionally coherent pattern of microbial response. The functional profiles inferred from PICRUSt2 were directionally consistent with this taxonomic pattern. LP-L showed a higher predicted abundance of KOs annotated as SCFA production, butanoate metabolism, bile acid metabolism, and anaerobic energy fermentation relative to all three comparators. These findings suggest that the taxonomic shifts observed in the low-diversity subgroup were accompanied by a coordinated shift in the predicted community-level metabolic capacity. These directional patterns are consistent with previous reports describing the reduced representation of SCFA- and butanoate-related pathways in obesity-associated dysbiosis.^12^ ^,^ ^13^ Alignment between the predicted functional profiles and taxonomic enrichment of butyrate-producing taxa such as *Faecalibacterium* and *Ruminococcus* supports the internal coherence of these findings. PICRUSt2-based inference represents the predicted metabolic potential rather than direct pathway activity and should be interpreted accordingly.^40^ These predictions describe the functional potential of the resident community and cannot be assigned specifically to LRCC5282. Nevertheless, previous studies integrating inference-based profiling with fecal metabolite measurements have reported a directional correspondence between predicted fermentation-related pathways and fecal SCFA concentrations.^63^ The agreement between the predicted functions and the measured fecal metabolites strengthens the internal consistency of the findings, but does not confirm pathway activation.

The gut microbiota modulates bile acid compositional profiles through sequential deconjugation by bile salt hydrolase (BSH)-expressing bacteria followed by 7α-dehydroxylation encoded by the *bai* operon. The abundance and enzymatic capacity of these bacteria collectively determine the primary-to-secondary bile acid conversion ratio and downstream FXR and TGR5 signaling pathways.^14^ *Ruminococcaceae* harbor distinctive secondary bile acid-producing enzymatic capacities, including unique iso-bile acid biosynthetic pathways.^64^ Members of the *Oscillospiraceae* family coharbor both the *bai* operon and BSH, facilitating sequential bile acid biotransformation.^65^ *Eubacterium* represents a well-characterized genus with established 7α-dehydroxylation capacity.^66^ The concurrent enrichment of these taxa in the low-diversity subgroup following LP5282-*P* administration, together with a significant increase in the proportion of cholic acid and reduction in lithocholic acid, glycocholic acid, and taurocholic acid, is consistent with altered community-level bile acid-transforming enzymatic activity. No between-group differences were observed in total bile acid concentrations. This supports the interpretation that these changes reflect alterations in microbial biotransformation patterns rather than absolute production capacity. No corresponding bile acid compositional differences were detected in the high-diversity subgroup, suggesting that bile acid responses may be conditioned by the baseline microbial characteristics. SCFAs are key microbial metabolites that mediate host metabolic regulation through colonocyte energy supply, gut barrier maintenance, immune modulation, and FFAR2/FFAR3-dependent signaling.^12^ Butyrate has been extensively studied for its role in maintaining the integrity of the epithelial barrier and immune homeostasis. The consistent depletion of butyrate-producing taxa and reduced representation of the butanoate pathway have been reported in obesity-associated dysbiosis.^57^ Acetate and propionate participate in broad metabolic networks. Propionate is principally utilized in hepatic gluconeogenesis, and associations between these SCFAs and host metabolic outcomes have been inconsistent across studies.^67^ In the low-diversity subgroup, significant elevations in acetate and butyrate levels were concordant with the enrichment of butyrate-producing taxa such as *Faecalibacterium* and *Ruminococcus*, and with a higher predicted abundance of butanoate metabolism-related KOs. No corresponding differences were observed in the high-diversity subgroup, indicating that the SCFA responses were conditioned by the baseline microbial characteristics. Propionate showed a divergent pattern relative to acetate and butyrate. Significant elevations were observed in both LP5282-*P* subgroups relative to the low-diversity placebo group. However, the concentrations of propionate were lower in the low-diversity treatment group than in the high-diversity treatment group, suggesting differences in fermentation substrate utilization across subgroups. Fecal SCFA concentrations reflect the net intestinal balance rather than microbial production alone. Therefore, these changes should be interpreted as indicators of altered microbiota-host metabolic equilibrium rather than direct evidence of increased fermentation output.^68^ Concurrent bile acid compositional shifts and SCFA elevations in the low-diversity subgroup indicated coordinated changes across multiple metabolite compartments following LP5282-*P* administration. This pattern was not observed in the high-diversity subgroups. The short-chain fatty acids and bile acids quantified in this study constitute only a subset of the bioactive metabolites produced by *L. plantarum*. Through its proteolytic system and amino acid metabolism, *L. plantarum* can yield bioactive peptides with antioxidant, antihypertensive, and lipid-regulating activities.^69^ It also generates *γ*-aminobutyric acid, which has been associated with improved lipid metabolism and gut microbiota modulation in obesity models.^70^ Its principal organic acids, lactic and acetic acid, also act as cross-feeding precursors for butyrate- and propionate-producing commensals.^13^ Aromatic amino acid-derived metabolites, particularly tryptophan-derived indoles formed through host–microbial co-metabolism, act as aryl hydrocarbon receptor ligands that reinforce gut barrier integrity, stimulate GLP-1 secretion, and improve insulin sensitivity.^71^ ^,^ ^72^ Consistent with the diversity-conditioned rationale and the prespecified microbiota outcome, the present mechanistic analysis focused on the resident microbiota and its community-level output, namely fecal short-chain fatty acids and bile acids. These strain-derived metabolites, including *γ*-aminobutyric acid, organic acids, bioactive peptides, and aromatic amino acid-derived metabolites, were not measured. Their targeted quantification would have permitted a more complete characterization of the metabolite profile underlying the observed responses, and remains a priority for future work to further define the therapeutic potential of LP5282-P.

Body weight and BMI are established indicators of obesity-related cardiometabolic risk and are independently associated with insulin resistance, dyslipidemia, and cardiovascular disease.^4^ DXA-derived total body fat mass provides a more precise quantification of adipose tissue accumulation than BMI alone and has shown stronger associations with cardiometabolic risk factors in multiple studies.^8^ In obesity, these indices are strongly correlated and tend to be clustered with other metabolic risk factors, reflecting the progressive nature of adiposity-related metabolic dysregulation.^4^ Leptin, an adipokine secreted by adipocytes in proportion to fat mass, regulates appetite and energy expenditure through the JAK2-STAT3 signaling pathway in hypothalamic neurons, and serves as a hormonal indicator of adipose tissue status.^73^ In obesity, sustained hyperleptinemia induces leptin resistance by impairing this signaling pathway. This disruption contributes to dysregulated appetite control, chronic low-grade inflammation, oxidative stress, and endothelial dysfunction.^74^ The relationship between the gut microbiota and leptin has been proposed to be bidirectional. A reduction in the abundances of *Faecalibacterium* and *Akkermansia* in obesity has been reported to co-occur with decreased microbial diversity and elevated leptin concentrations across multiple human cohort studies.^75^ Moreover, gut microbiota-derived metabolites have been proposed to modulate leptin sensitivity via the JAK2-STAT3 pathway.^76^ The significant treatment-by-diversity interactions observed for body weight, BMI, leptin, and subcutaneous fat area, which were restricted to the low-diversity treatment group, indicate that these clinical responses were associated with the interaction between treatment assignment and baseline microbial characteristics rather than treatment alone. This interaction pattern supports the concept that clinical responsiveness to microbiome-targeted interventions varies according to pre-existing microbial characteristics. Therefore, baseline microbial stratification may be required to identify individuals in whom such interventions can elicit detectable clinical effects.^20^

The correlation pattern observed in the low-diversity subgroup supports a biologically coherent framework relating changes in the resident microbiota to fecal metabolites and clinical outcomes. The increased abundances of butyrate-associated taxa, including *Faecalibacterium, Ruminococcus*, and *Eubacterium*, paralleled the increase in fecal butyrate concentrations. Butyrate has been reported to reinforce intestinal epithelial barrier integrity through upregulation of tight junction proteins ZO-1, occludin, and claudin-3 and through activation of the AMPK/PGC-1α pathway, thereby limiting the systemic translocation of LPS.^77^ ^,^ ^78^ *A. muciniphila*, through its outer membrane protein Amuc_1100, has been reported to strengthen gut barrier function via TLR2 activation, increasing mucus layer thickness and tight junction protein expression.^25^ ^,^ ^79^ Sustained LPS-mediated TLR4/NF-κB signaling in adipose tissue promotes macrophage infiltration, chronic low-grade inflammation, and adipose tissue expansion.^1^ ^,^ ^6^ ^,^ ^80^ Conversely, improvement in intestinal barrier function has been associated with reduced systemic LPS concentrations and attenuation of adipose inflammation.^81^ The levels of leptin, an adipokine that integrates adipose tissue mass and inflammatory status, may decline in response to the concurrent suppression of adipose tissue expansion and inflammation.^73^ ^,^ ^74^ These pathways offer biological plausibility for the inverse correlations of *Akkermansia* and *Eubacterium* with body weight, body fat mass, and leptin, and of *Oscillibacter* with leptin. Butyrate has also been reported to stimulate lipolysis in white adipose tissue through histone deacetylase inhibition and downstream cAMP–PKA signaling and to increase adiponectin receptor expression,^82^ which provides a biological basis for the positive correlation between *Faecalibacterium* and adiponectin. In addition, activation of TGR5 enhances thermogenesis and energy expenditure and promotes anti-inflammatory macrophage polarization in adipose tissue,^83^ offering a plausible context for the association between bile acid composition and adiposity-related outcomes. Consistent with this bile acid–centered interpretation, *Parabacteroides* was inversely correlated with BMI and body fat mass, a pattern consistent with its reported production of succinate and secondary bile acids and with the inverse association between its abundance and BMI observed in human cohorts.^84^ The Shannon and Chao1 indices showed correlations with the fecal metabolites and clinical outcomes that were consistent in direction with the taxon-level associations; higher diversity was associated with increased fecal short-chain fatty acid concentrations, decreased deoxycholic acid, and reductions in body weight and leptin. This pattern is in keeping with prior evidence that gut microbial richness is diminished in individuals with greater adiposity and metabolic impairment^7^ and across higher body mass index categories,^85^ that baseline diversity is predictive of the metabolic response to microbiota-targeted obesity interventions,^33^ and that fecal short-chain fatty acid concentrations are positively associated with microbial diversity and are higher in non-obese than in obese individuals.^86^ ^,^ ^87^ The community-level indices therefore aligned with the same metabolic and clinical axis as the responsive taxa, indicating that the favorable changes within the low-diversity subgroup were captured at the level of overall community diversity as well as that of individual genera. These correlations, however, do not establish temporal sequence, mediation, or causality.

The composite *α*-diversity score used for stratification summarizes community richness and evenness but does not resolve which taxa dominate the resident microbiota. Compositional structure, conventionally represented as enterotypes characterized by the predominance of *Bacteroides, Prevotella*, or *Ruminococcus*, constitutes a distinct descriptor. These enterotypes are associated with divergent fermentation substrate preferences and short-chain fatty acid profiles.^88^ The relevance of this compositional axis to intervention responsiveness is supported by *in vitro* evidence that soluble *β*-glucan selectively enriches *Bacteroides* and suppresses *Prevotella*, with donor-dependent propionate and butyrate production, indicating that the relative representation of *Bacteroides* and *Prevotella* may underlie inter-individual differences in fermentation.^89^ Consistent with this, the taxa correlated with the metabolite and clinical responses in the present low-diversity subgroup comprised both enterotype-defining and short-chain fatty acid-producing genera, and the LP5282-*P*-associated enrichment of *Bacteroidota* in this subgroup is concordant with a shift along this compositional axis. The two-group stratification adopted here corresponds to the diversity-based premise of the study and retains subgroup sizes adequate for the within-subgroup analyses. An enterotype-based classification would further resolve compositional features not reflected in *α*-diversity alone. The two approaches are therefore complementary, and their concurrent evaluation in larger cohorts may contribute to the identification of paraprobiotic responders.

This study has some limitations that warrant consideration when interpreting the findings. The absence of significant treatment effects in the overall PP population indicates that the clinical utility of LP5282-*P* as a generalized metabolic intervention remains limited. Therefore, the subgroup-level responses observed in the present study require prospective validation in independently designed trials with pre-specified diversity-based stratification. Co-administration with targeted prebiotics or the identification of additional bioactive constituents derived from LP5282-*P* may represent a viable strategy for extending its modulatory capacity across a broader spectrum of baseline microbial configurations.^90^ However, these strategies require further investigation in future studies. In addition, quantitative dietary intake was not measured. Although the three-day dietary records showed no differences across subgroups, future studies incorporating objective dietary assessment would more definitively exclude dietary confounding. The trial did not include strain-specific measures of intervention engagement. The V3–V4 amplicon approach did not enable strain-specific detection or tracking of LRCC5282, and no strain-associated metabolites or proximal host-response markers were assessed. The changes were confined to the LP5282-*P* subgroup and were not observed in placebo. However, the absence of strain-specific exposure indicators means these findings cannot be assigned to a strain-specific molecular mechanism, and the present design does not permit formal testing of their mediating role in the clinical responses. Host-response markers corresponding to strain-specific exposure were likewise not assessed. Plasma LPS, intestinal permeability, and circulating bile acid receptor-associated hormones such as FGF19 and GLP-1 were not measured, nor were downstream metabolites of the fecal bile acid and SCFA compartments. In addition, strain-specific biomarkers and functional validation will be needed to establish a more direct mechanistic link between LP5282-*P* and the observed host responses. Complementary metagenomic and metatranscriptomic profiling can further address the resolution constraints inherent in 16S rRNA amplicon sequencing. Such approaches would enable a more precise functional characterization of the LP5282-*P*-associated compositional shifts identified in this study. Moreover, the durability of the observed responses beyond the 12-week intervention period was not evaluated. The extent to which the restructured microbial community configuration, associated metabolites, and clinical changes persist after LP5282-*P* discontinuation has direct implications for its clinical utility and the design of subsequent intervention studies incorporating structured post-intervention follow-up assessments.

## Conclusions

The present study evaluated the clinical, microbiome, and metabolite responsiveness to LP5282-*P* in overweight adults. Stratified analyses based on baseline gut microbial diversity were applied to determine whether pre-existing microbial community characteristics conditioned paraprobiotic responsiveness. Across the overall PP population, no significant between-group differences were observed for any clinical outcome. In contrast, participants with low baseline gut microbial diversity demonstrated significant and coherent responses across the clinical, microbiome, and fecal metabolite outcome domains, whereas those with high baseline diversity showed only isolated, non-coherent differences. These findings indicate that baseline gut microbial diversity may condition the response to LP5282-*P*, as evidenced by coordinated changes in clinical, microbial, and metabolic outcomes observed primarily among participants with low baseline diversity. Therefore, baseline microbiome diversity profiling may be a potential stratification marker to improve responder identification in future microbiota-targeted interventional trials. Prospective studies that directly assess intervention engagement and host–microbiome function, coupled with compositional stratification approaches such as enterotyping, are needed to clarify the clinical relevance of this diversity-dependent response and to provide a more direct mechanistic basis for it.

## Supplementary Material

Supplementary Material Supplementary_Fig2.tif

Supplementary Material Supplementary file clean.docx

Supplementary Material Supplementary_Fig1.tif

## Data Availability

The raw 16S rRNA gene sequencing data generated in this study has been deposited in the NCBI Sequence Read Archive (SRA) under the BioProject accession number PRJNA1404593 (https://www.ncbi.nlm.nih.gov/bioproject/PRJNA1404593). The data are accessible via the NCBI SRA submission SUB15958216 (https://dataview.ncbi.nlm.nih.gov/?search=SUB15958216&archive=sra) and comprise the individual SRA run accession numbers SRR36969092–SRR36969293.

## Abbreviations

ANCOM-BC: Analysis of Composition of Microbiomes with Bias Correction
CRIS: Clinical Research Information Service
CT: Computed tomography
DXA: Dual-energy X-ray absorptiometry
FA set: Full analysis set
F/B ratio: *Firmicutes*-to-*Bacteroidota* ratio
hs-CRP: High-sensitivity C-reactive protein
ICH-GCP: International Council for Harmonization-Good Clinical Practice
KEGG: Kyoto Encyclopedia of Genes and Genomes
KO: KEGG ortholog
LEfSe: Linear discriminant analysis effect size
LP-BL: LP5282-P group at baseline
LP-H: High-diversity LP5282-P subgroup
LP-L: Low-diversity LP5282-P subgroup
LP-12: LP5282-P group at week 12
LP5282-P: Paraprobiotics derived from *L. plantarum* LRCC5282
LRCC: LOTTE R&D Culture Collection
MaAsLin2: Multivariable Association with Linear Models 2
PCoA: Principal coordinates analysis
PICRUSt2: Phylogenetic Investigation of Communities by Reconstruction of Unobserved States 2
PL-BL: Placebo group at baseline
PL-H: High-diversity placebo subgroup
PL-L: Low-diversity placebo subgroup
PL-12: Placebo group at week 12
PP set: Per-protocol set
SCFA: Short-chain fatty acid
16S rRNA: 16S ribosomal RNA gene
