# Supplementary material for: Gut microbial diversity at baseline conditions the clinical, microbiome, and metabolic response to paraprobiotic Lactiplantibacillus plantarum LRCC5282 in overweight adults
Source: Gut Microbes. 2026 Aug 25;18(1):2722835. doi: 10.1080/19490976.2026.2722835 (PMC13523931; doi:10.1080/19490976.2026.2722835)
Supplement: Supplementary file 1 [file KGMI_A_2722835_SM1308.docx]

**Supplementary Tables**

**Supplementary Table 1.** Baseline characteristics of participants in the per-protocol population

| Characteristic | Placebo  (n = 52) | LP5282-P  (n = 49) | P-value |  |
| --- | --- | --- | --- | --- |
| Sex, n (%) |  |  | 0.5203 |  |
| - Male | 18 (34.62) | 20 (40.82) |  |  |
| - Female | 34 (65.38) | 29 (59.18) |  |  |
| Reproductive status  (females only), n (%) |  |  | 0.9549 |  |
| - Yes | 34 (76.47) | 29 (75.86) |  |  |
| - No | 8 (23.53) | 7 (24.14) |  |  |
| Age (years) | 43.23 ± 10.56 | 43.80 ± 9.74 | 0.7807 |  |
| Height (cm) | 163.66 ± 8.39 | 165.38 ± 8.35 | 0.2739 |  |
| Body weight (kg) | 72.84 ± 8.10 | 73.50 ± 8.80 | 0.8015 |  |
| BMI (kg/m²) | 27.13 ± 1.37 | 26.80 ± 1.39 | 0.1894 |  |
| Current alcohol consumption,  n (%) |  |  | 0.6166 |  |
| - Yes | 27 (51.92) | 23 (46.94) |  |  |
| - No | 25 (48.08) | 26 (53.06) |  |  |
| Smoking status, n (%) |  |  | 0.3739 |  |
| - Current smoker | 6 (11.54) | 6 (12.24) |  |  |
| - former or never | 46 (88.46) | 43 (87.76) |  |  |
| Comorbid conditions, n (%) |  |  | 0.3748 |  |
| - Present | 23 (44.23) | 26 (53.06) |  |  |
| - Absent | 29 (55.77) | 23 (46.94) |  |  |
| Concomitant medication use,  n (%) |  |  | 0.8400 |  |
| - Yes | 24 (46.15) | 24 (48.98) |  | |
| - No | 28 (53.85) | 25 (51.02) |  |  |

BMI, body mass index; LP5282-P, paraprobiotic derived from *Lactiplantibacillus plantarum* LRCC5282; n, number of participants included in the per-protocol analysis from each group.

Data are presented as mean ± SEM or n (%). P-values represent between-group comparisons. P < 0.05 was considered statistically significant.

**Supplementary Table 2.** Hematology and serum chemistry parameters in the per-protocol population from screening to week 12.

| Contents | Placebo (n = 52) | LP5282-P (n = 49) | P-value |
| --- | --- | --- | --- |
| Albumin, g/dL | |  |  |
| - visit 1 (screening) | 4.62 ± 0.04 | 4.61 ± 0.04 | 0.8472 |
| - visit 4 (12-wks) | 4.61 ± 0.04 | 4.58 ± 0.05 | 0.6796 |
| - changes from baseline | -0.12 ± 0.03 | -0.03 ± 0.02 | 0.7306 |
| Alkaline phosphatase (ALP), IU/L | |  |  |
| - visit 1 (screening) | 62.83 ± 2.32 | 63.10 ± 2.40 | 0.9344 |
| - visit 4 (12-wks) | 65.50 ± 2.45 | 66.82 ± 2.85 | 0.7268 |
| - changes from baseline | 2.67 ± 1.27 | 3.71 ± 1.58 | 0.6080 |
| Alanine aminotransferase (ALT), IU/L | |  |  |
| - visit 1 (screening) | 23.35 ± 2.65 | 22.31 ± 1.79 | 0.7460 |
| - visit 4 (12-wks) | 22.06 ± 1.85 | 22.20 ± 1.68 | 0.9535 |
| - changes from baseline | -1.29 ± 2.18 | 0.10 ± 1.51 | 0.6018 |
| Aspartate aminotransferase (AST), IU/L | |  |  |
| - visit 1 (screening) | 23.92 ± 1.33 | 23.47 ± 1.53 | 0.8232 |
| - visit 4 (12-wks) | 23.00 ± 1.18 | 23.08 ± 0.92 | 0.9566 |
| - changes from baseline | -0.92 ± 0.90 | -0.39 ± 1.29 | 0.7341 |
| Basophil, % | |  |  |
| - visit 1 (screening) | 0.72 ± 0.05 | 0.62 ± 0.04 | 0.1276 |
| - visit 4 (12-wks) | 0.68 ± 0.05 | 0.64 ± 0.04 | 0.5360 |
| - changes from baseline | -0.03 ± 0.04 | 0.02 ± 0.03 | 0.2328 |
| Blood urea nitrogen (BUN), mg/dL | |  |  |
| - visit 1 (screening) | 12.70 ± 0.51 | 12.91 ± 0.46 | 0.7578 |
| - visit 4 (12-wks) | 12.36 ± 0.48 | 12.85 ± 0.43 | 0.4473 |
| - changes from baseline | -0.34 ± 0.53 | -0.37 ± 0.49 | 0.9701 |
| Calcium (Ca), mmol/L | |  |  |
| - visit 1 (screening) | 9.42 ± 0.05 | 9.39 ± 0.05 | 0.6924 |
| - visit 4 (12-wks) | 9.41 ± 0.05 | 9.42 ± 0.06 | 0.9086 |
| - changes from baseline | -0.00 ± 0.05 | 0.04 ± 0.05 | 0.5676 |
| Creatine kinase (CK), IU/L | |  |  |
| - visit 1 (screening) | 200.70 ± 50.31 | 170.60 ± 31.81 | 0.6137 |
| - visit 4 (12-wks) | 149.00 ± 19.94 | 137.70 ± 17.22 | 0.6694 |
| - changes from baseline | -51.71 ± 44.52 | -32.84 ± 32.54 | 0.7329 |
| Chloride (Cl), mmol/L | |  |  |
| - visit 1 (screening) | 104.10 ± 0.28 | 104.30 ± 0.30 | 0.5867 |
| - visit 4 (12-wks) | 103.90 ± 0.25 | 104.40 ± 0.31 | 0.2663 |
| - changes from baseline | -0.13 ± 0.30 | 0.10 ± 0.35 | 0.6310 |
| Creatinine, mg/dL | |  |  |
| - visit 1 (screening) | 0.76 ± 0.02 | 0.75 ± 0.02 | 0.9445 |
| - visit 4 (12-wks) | 0.75 ± 0.02 | 0.78 ± 0.03 | 0.4041 |
| - changes from baseline | -0.01 ± 0.01 | 0.02 ± 0.01 | 0.0797 |
| Eosinophil, % | |  |  |
| - visit 1 (screening) | 2.52 ± 0.31 | 2.08 ± 0.22 | 0.2511 |
| - visit 4 (12-wks) | 2.33 ± 0.27 | 1.89 ± 0.17 | 0.1677 |
| - changes from baseline | -0.19 ± 0.17 | -0.19 ± 0.14 | 0.9983 |
| Glucose, mg/dL | |  |  |
| - visit 1 (screening) | 100.30 ± 1.45 | 98.51 ± 1.35 | 0.3768 |
| - visit 4 (12-wks) | 95.75 ± 1.82 | 96.35 ± 1.31 | 0.7911 |
| - changes from baseline | -4.52 ± 1.67 | -2.16 ± 1.09 | 0.2407 |
| γ-glutamyl transferase (GTP), IU/L | |  |  |
| - visit 1 (screening) | 26.94 ± 2.73 | 24.86 ± 1.71 | 0.5192 |
| - visit 4 (12-wks) | 27.94 ± 3.59 | 27.22 ± 2.89 | 0.8765 |
| - changes from baseline | 1.00 ± 1.57 | 2.37 ± 2.12 | 0.6051 |
| Hemoglobin (Hb), g/dL | |  |  |
| - visit 1 (screening) | 13.93 ± 0.20 | 14.08 ± 0.19 | 0.5931 |
| - visit 4 (12-wks) | 13.88 ± 0.20 | 14.22 ± 0.20 | 0.2282 |
| - changes from baseline | -0.05 ± 0.09 | 0.14 ± 0.09 | 0.1208 |
| Hematocrit (Hct), % | |  |  |
| - visit 1 (screening) | 42.27 ± 0.53 | 42.62 ± 0.47 | 0.6237 |
| - visit 4 (12-wks) | 42.07 ± 0.52 | 42.93 ± 0.55 | 0.2615 |
| - changes from baseline | -0.20 ± 0.24 | 0.31 ± 0.30 | 0.1880 |
| Potassium (K), mmol/L | |  |  |
| - visit 1 (screening) | 4.28 ± 0.04 | 4.31 ± 0.04 | 0.5604 |
| - visit 4 (12-wks) | 4.31 ± 0.04 | 4.29 ± 0.04 | 0.7391 |
| - changes from baseline | 0.03 ± 0.04 | -0.02 ± 0.04 | 0.3817 |
| Lymphocyte, % | |  |  |
| - visit 1 (screening) | 34.72 ± 0.96 | 35.06 ± 1.02 | 0.8106 |
| - visit 4 (12-wks) | 33.81 ± 0.89 | 35.64 ± 1.04 | 0.1826 |
| - changes from baseline | -0.92 ± 0.94 | 0.58 ± 0.89 | 0.2511 |
| Mean corpuscular volume (MCV), fL | |  |  |
| - visit 1 (screening) | 90.44 ± 0.63 | 89.69 ± 0.61 | 0.3938 |
| - visit 4 (12-wks) | 90.45 ± 0.63 | 89.88 ± 0.62 | 0.5185 |
| - changes from baseline | 0.01 ± 0.24 | 0.18 ± 0.23 | 0.5959 |
| Monocyte, % | |  |  |
| - visit 1 (screening) | 6.66 ± 0.21 | 6.92 ± 0.22 | 0.3924 |
| - visit 4 (12-wks) | 6.90 ± 0.26 | 7.20 ± 0.27 | 0.4227 |
| - changes from baseline | 0.23 ± 0.22 | 0.28 ± 0.18 | 0.8861 |
| Sodium (Na), mmol/L | |  |  |
| - visit 1 (screening) | 140.30 ± 0.31 | 140.00 ± 0.33 | 0.6076 |
| - visit 4 (12-wks) | 140.30 ± 0.26 | 140.30 ± 0.27 | 0.8708 |
| - changes from baseline | -0.02 ± 0.32 | 0.28 ± 0.31 | 0.5093 |
| Neutrophil, % | |  |  |
| - visit 1 (screening) | 55.32 ± 1.03 | 55.30 ± 1.10 | 0.9897 |
| - visit 4 (12-wks) | 56.16 ± 0.98 | 54.51 ± 1.16 | 0.2784 |
| - changes from baseline | 0.84 ± 1.03 | 0.78 ± 1.05 | 0.2700 |
| Platelet (PLT), 10^3^/μL | |  |  |
| - visit 1 (screening) | 283.70 ± 8.14 | 273.50 ± 7.84 | 0.3690 |
| - visit 4 (12-wks) | 284.40 ± 8.51 | 276.80 ± 8.27 | 0.5209 |
| - changes from baseline | 0.71 ± 2.85 | 3.27 ± 5.80 | 0.6940 |
| Protein, g/dL | |  |  |
| - visit 1 (screening) | 7.35 ± 0.06 | 7.42 ± 0.05 | 0.3323 |
| - visit 4 (12-wks) | 7.40 ± 0.05 | 7.46 ± 0.05 | 0.3891 |
| - changes from baseline | 0.06 ± 0.05 | 0.04 ± 0.04 | 0.8051 |
| Red blood cell (RBC), 10^6^/μL | |  |  |
| - visit 1 (screening) | 4.68 ± 0.06 | 4.76 ± 0.06 | 0.3397 |
| - visit 4 (12-wks) | 4.65 ± 0.06 | 4.79 ± 0.07 | 0.1318 |
| - changes from baseline | -0.03 ± 0.03 | 0.02 ± 0.03 | 0.2100 |
| Total bilirubin, g/dL | |  |  |
| - visit 1 (screening) | 0.66 ± 0.05 | 0.65 ± 0.04 | 0.8776 |
| - visit 4 (12-wks) | 0.68 ± 0.04 | 0.70 ± 0.04 | 0.7778 |
| - changes from baseline | 0.02 ± 0.04 | 0.04 ± 0.04 | 0.6451 |
| White blood cell (WBC), 10^3^/μL | |  |  |
| - visit 1 (screening) | 6.07 ± 0.17 | 5.89 ± 0.17 | 0.4554 |
| - visit 4 (12-wks) | 6.16 ± 0.16 | 5.84 ± 0.15 | 0.1534 |
| - changes from baseline | 0.09 ± 0.14 | -0.05 ± 0.16 | 0.5195 |

LP5282-P, paraprobiotic derived from *Lactiplantibacillus plantarum* LRCC5282; n, number of participants included in the per-protocol analysis from each group.

Data are presented as mean ± SEM. P-values represent between-group comparisons, with P < 0.05 considered statistically significant.

**Supplementary Table 3.** Baseline characteristics of participants in the high-diversity subgroup.

| Characteristic | Placebo (High-diversity, n = 26) | LP5282-P (High-diversity, n = 25) | P-value |
| --- | --- | --- | --- |
| Sex, n (%) |  |  | 0.8199 |
| - Male | 8 (30.77) | 6 (24.00) |  |
| - Female | 18 (69.23) | 19 (76.00) |  |
| Reproductive status  (females only), n (%) |  |  | 0.9257 |
| - Yes | 13 (77.22) | 15 (78.95) |  |
| - No | 5 (27.78) | 4(21.05) |  |
| Age (years) | 43.92 ± 2.25 | 45.52 ± 2.04 | 0.6019 |
| Height (cm) | 162.01 ± 1.34 | 162.63 ± 1.21 | 0.7568 |
| Body weight (kg) | 71.32 ± 1.53 | 70.39 ± 1.05 | 0.6214 |
| BMI (kg/m²) | 27.10 ± 0.28 | 26.60 ± 0.24 | 0.1872 |
| Current alcohol consumption, n (%) |  |  | 0.8616 |
| - Yes | 11 (42.31) | 9 (36.00) |  |
| - No | 15 (57.69) | 16 (64.00) |  |
| Smoking status, n (%) |  |  | 0.7013 |
| - Current smoker | 4 (15.38) | 2 (8.00) |  |
| - former or never | 22 (84.62) | 23 (92.00) |  |
| Comorbid conditions, n (%) |  |  | 0.8990 |
| - Present | 15 (57.69) | 13 (52.00) |  |
| - Absent | 11 (42.31) | 12 (48.00) |  |
| Concomitant medication use, n (%) |  |  | 0.9141 |
| - Yes | 10 (38.46) | 9 (36.00) |  |
| - No | 16 (61.54) | 16 (64.00) |  |
| IP (%) | 96.89±1.28 | 98.13±1.74 | 0.5728 |

BMI, body mass index; IP, investigational product compliance; LP5282-P, paraprobiotic derived from *Lactiplantibacillus plantarum* LRCC5282; n, number of participants included in the per-protocol analysis from each high-diversity group.

Data are presented as mean ± SEM or n (%). P-values represent between-group comparisons, with P < 0.05 considered statistically significant.

**Supplementary Table 4.** Baseline characteristics of participants in the low-diversity subgroup.

| Characteristic | Placebo (Low-diversity, n = 26) | LP5282-P (Low-diversity, n = 24) | P-value |
| --- | --- | --- | --- |
| Sex, n (%) |  |  | 0.3958 |
| - Male | 11 (42.31) | 14 (58.33) |  |
| - Female | 15 (57.69) | 10 (41.67) |  |
| Reproductive status  (females only), n (%) |  |  | 0.9238 |
| - Yes | 12 (80.00) | 7 (70.00) |  |
| - No | 3 (20.00) | 3 (30.00) |  |
| Age (years) | 42.69 ± 1.96 | 41.48 ± 1.86 | 0.6554 |
| Height (cm) | 165.51 ± 1.84 | 168.33 ± 1.94 | 0.3037 |
| Body weight (kg) | 74.02 ± 1.63 | 76.85 ± 2.11 | 0.2864 |
| BMI (kg/m²) | 26.96 ± 0.25 | 27.01 ± 0.29 | 0.9120 |
| Current alcohol consumption, n (%) |  |  | 0.9539 |
| - Yes | 16 (61.54) | 14 (58.53) |  |
| - No | 10 (38.46) | 10 (41.67) |  |
| Smoking status, n (%) |  |  | 0.6203 |
| - Current smoker | 4 (15.38) | 6 (25.00) |  |
| - former or never | 22 (84.62) | 18 (75.00) |  |
| Comorbid conditions, n (%) |  |  | 0.7394 |
| - Present | 12 (46.15) | 9 (37.50) |  |
| - Absent | 14 (53.85) | 15 (62.50) |  |
| Concomitant medication use, n (%) |  |  | 0.5919 |
| - Yes | 16 (61.54) | 12 (50.00) |  |
| - No | 10 (38.46) | 12 (50.00) |  |
| IP (%) | 99.84±1.58 | 99.79±1.93 | 0.9832 |

BMI, body mass index; IP, investigational product compliance; LP5282-P, paraprobiotic derived from *Lactiplantibacillus plantarum* LRCC5282; n, number of participants included in the per-protocol analysis from each low-diversity subgroup.

Data are presented as mean ± SEM or n (%). P-values represent between-group comparisons, with P < 0.05 considered statistically significant.

**Supplementary Table 5.** Hematology and serum chemistry parameters in the high-diversity subgroup from screening to week 12

| Contents | Placebo (High-diversity, n = 26) | LP5282-P (High-diversity, n = 25) | P-value |
| --- | --- | --- | --- |
| Albumin, g/dL | |  |  |
| - visit 1 (screening) | 4.66 ± 0.05 | 4.56 ± 0.05 | 0.1457 |
| - visit 4 (12-wks) | 4.57 ± 0.04 | 4.52 ± 0.07 | 0.5693 |
| - changes from baseline | -0.10 ± 0.04 | -0.04 ± 0.04 | 0.2842 |
| Alkaline phosphatase (ALP), IU/L | |  |  |
| - visit 1 (screening) | 66.38 ± 3.24 | 65.28 ± 3.31 | 0.8124 |
| - visit 4 (12-wks) | 66.30 ± 3.29 | 71.00 ± 3.74 | 0.3458 |
| - changes from baseline | -0.12 ± 1.70 | 5.72 ± 2.43 | 0.0630 |
| Alanine aminotransferase (ALT), IU/L | |  |  |
| - visit 1 (screening) | 25.35 ± 4.60 | 19.24 ± 2.04 | 0.2365 |
| - visit 4 (12-wks) | 21.81 ± 2.12 | 20.84 ± 2.49 | 0.7680 |
| - changes from baseline | -3.54 ± 4.14 | 1.60 ± 1.86 | 0.2690 |
| Aspartate aminotransferase (AST), IU/L | |  |  |
| - visit 1 (screening) | 23.81 ± 2.02 | 21.68 ± 1.52 | 0.4064 |
| - visit 4 (12-wks) | 22.23 ± 1.36 | 22.52 ± 1.30 | 0.8785 |
| - changes from baseline | -1.58 ± 1.53 | 0.84 ± 1.59 | 0.2793 |
| Basophil, % | |  |  |
| - visit 1 (screening) | 0.72 ± 0.07 | 0.69 ± 0.07 | 0.8085 |
| - visit 4 (12-wks) | 0.73 ± 0.07 | 0.72 ± 0.06 | 0.8750 |
| - changes from baseline | 0.01 ± 0.06 | 0.02 ± 0.06 | 0.9167 |
| Blood urea nitrogen (BUN), mg/dL | |  |  |
| - visit 1 (screening) | 13.12 ± 0.88 | 12.28 ± 0.54 | 0.4242 |
| - visit 4 (12-wks) | 12.80 ± 0.64 | 12.52 ± 0.59 | 0.7531 |
| - changes from baseline | -0.32 ± 0.83 | 0.24 ± 0.60 | 0.5867 |
| Calcium (Ca), mmol/L | |  |  |
| - visit 1 (screening) | 9.49 ± 0.08 | 9.33 ± 0.07 | 0.1467 |
| - visit 4 (12-wks) | 9.40 ± 0.08 | 9.31 ± 0.09 | 0.4744 |
| - changes from baseline | -0.08 ± 0.09 | -0.02 ± 0.06 | 0.5311 |
| Creatine kinase (CK), IU/L | |  |  |
| - visit 1 (screening) | 230.62 ± 98.44 | 175.68 ± 58.06 | 0.6358 |
| - visit 4 (12-wks) | 149.80 ± 30.61 | 123.81 ± 20.96 | 0.4892 |
| - changes from baseline | -80.81 ± 84.13 | -51.84 ± 59.21 | 0.7810 |
| Chloride (Cl), mmol/L | |  |  |
| - visit 1 (screening) | 104.23 ± 0.48 | 104.42 ± 0.45 | 0.7166 |
| - visit 4 (12-wks) | 104.22 ± 0.32 | 104.43 ± 0.41 | 0.6893 |
| - changes from baseline | 0.01 ± 0.49 | 0.00 ± 0.51 | 0.9610 |
| Creatinine, mg/dL | |  |  |
| - visit 1 (screening) | 0.76 ± 0.12 | 0.70 ± 0.03 | 0.1206 |
| - visit 4 (12-wks) | 0.75 ± 0.03 | 0.72 ± 0.03 | 0.4370 |
| - changes from baseline | -0.00 ± 0.01 | 0.02 ± 0.02 | 0.2320 |
| Eosinophil, % | |  |  |
| - visit 1 (screening) | 2.30 ± 0.40 | 2.30 ± 0.31 | 0.9998 |
| - visit 4 (12-wks) | 2.12 ± 0.39 | 2.03 ± 0.23 | 0.8502 |
| - changes from baseline | -0.18 ± 0.18 | -0.26 ± 0.24 | 0.7667 |
| Glucose, mg/dL | |  |  |
| - visit 1 (screening) | 100.33 ± 2.09 | 98.84 ± 1.49 | 0.5825 |
| - visit 4 (12-wks) | 94.96 ± 3.04 | 95.28 ± 1.64 | 0.9277 |
| - changes from baseline | -5.31 ± 2.64 | -3.56 ± 1.49 | 0.5708 |
| γ-glutamyl transferase (GTP), IU/L | |  |  |
| - visit 1 (screening) | 24.27 ± 2.83 | 22.12 ± 2.56 | 0.5767 |
| - visit 4 (12-wks) | 26.58 ± 4.72 | 26.00 ± 5.12 | 0.9342 |
| - changes from baseline | 2.31 ± 2.54 | 3.88 ± 3.86 | 0.7330 |
| Hemoglobin (Hb), g/dL | |  |  |
| - visit 1 (screening) | 13.79 ± 0.31 | 13.74 ± 0.27 | 0.9067 |
| - visit 4 (12-wks) | 13.67 ± 0.31 | 13.87 ± 0.31 | 0.6437 |
| - changes from baseline | -0.12 ± 0.10 | 0.13 ± 0.12 | 0.1238 |
| Hematocrit (Hct), % | |  |  |
| - visit 1 (screening) | 41.92 ± 0.82 | 41.72 ± 0.68 | 0.8535 |
| - visit 4 (12-wks) | 41.68 ± 0.83 | 41.90 ± 0.85 | 0.8358 |
| - changes from baseline | -0.24 ± 0.27 | 0.18 ± 0.43 | 0.4096 |
| Potassium (K), mmol/L | |  |  |
| - visit 1 (screening) | 4.34 ± 0.04 | 4.31 ± 0.06 | 0.6633 |
| - visit 4 (12-wks) | 4.38 ± 0.04 | 4.32 ± 0.06 | 0.4113 |
| - changes from baseline | 0.04 ± 0.05 | 0.01 ± 0.05 | 0.7118 |
| Lymphocyte, % | |  |  |
| - visit 1 (screening) | 33.88 ± 1.38 | 34.93 ± 1.04 | 0.5501 |
| - visit 4 (12-wks) | 34.51 ± 1.31 | 35.63 ± 1.02 | 0.5027 |
| - changes from baseline | 0.63 ± 1.39 | 0.70 ± 1.21 | 0.9668 |
| Mean corpuscular volume (MCV), fL | |  |  |
| - visit 1 (screening) | 89.72 ± 0.96 | 90.82 ± 0.82 | 0.3897 |
| - visit 4 (12-wks) | 90.04 ± 0.98 | 91.04 ± 0.83 | 0.4466 |
| - changes from baseline | 0.33 ± 0.34 | 0.22 ± 0.34 | 0.8266 |
| Monocyte, % | |  |  |
| - visit 1 (screening) | 6.15 ± 0.30 | 6.78 ± 0.27 | 0.1253 |
| - visit 4 (12-wks) | 6.52 ± 0.29 | 6.96 ± 0.30 | 0.2962 |
| - changes from baseline | 0.37 ± 0.28 | 0.18 ± 0.25 | 0.6147 |
| Sodium (Na), mmol/L | |  |  |
| - visit 1 (screening) | 140.82 ± 0.48 | 139.74 ± 0.42 | 0.1180 |
| - visit 4 (12-wks) | 140.43 ± 0.28 | 140.16 ± 0.39 | 0.6138 |
| - changes from baseline | -0.38 ± 0.49 | 0.39 ± 0.43 | 0.2438 |
| Neutrophil, % | |  |  |
| - visit 1 (screening) | 56.90 ± 1.45 | 55.30 ± 1.11 | 0.3876 |
| - visit 4 (12-wks) | 55.99 ± 1.36 | 54.49 ± 1.14 | 0.4031 |
| - changes from baseline | -0.92 ± 1.49 | -0.82 ± 1.46 | 0.9624 |
| Platelet (PLT), 10^3^/μL | |  |  |
| - visit 1 (screening) | 291.13 ± 11.18 | 276.82 ± 10.53 | 0.3592 |
| - visit 4 (12-wks) | 291.32 ± 12.46 | 277.17 ± 9.42 | 0.3723 |
| - changes from baseline | 0.19 ± 3.31 | 0.28 ± 5.05 | 0.9884 |
| Protein, g/dL | |  |  |
| - visit 1 (screening) | 7.39 ± 0.08 | 7.34 ± 0.06 | 0.6410 |
| - visit 4 (12-wks) | 7.37 ± 0.07 | 7.43 ± 0.07 | 0.5379 |
| - changes from baseline | -0.02 ± 0.07 | 0.08 ± 0.05 | 0.2101 |
| Red blood cell (RBC), 10^6^/μL | |  |  |
| - visit 1 (screening) | 4.67 ± 0.08 | 4.60 ± 0.07 | 0.4779 |
| - visit 4 (12-wks) | 4.63 ± 0.08 | 4.61 ± 0.09 | 0.8400 |
| - changes from baseline | -0.05 ± 0.04 | 0.01 ± 0.04 | 0.3584 |
| Total bilirubin, g/dL | |  |  |
| - visit 1 (screening) | 0.69 ± 0.09 | 0.58 ± 0.04 | 0.2805 |
| - visit 4 (12-wks) | 0.66 ± 0.07 | 0.65 ± 0.04 | 0.8976 |
| - changes from baseline | -0.03 ± 0.07 | 0.07 ± 0.05 | 0.2516 |
| White blood cell (WBC), 10^3^/μL | |  |  |
| - visit 1 (screening) | 6.10 ± 0.22 | 6.04 ± 0.28 | 0.7916 |
| - visit 4 (12-wks) | 6.13 ± 0.24 | 5.94 ± 0.19 | 0.5346 |
| - changes from baseline | 0.03 ± 0.22 | -0.07 ± 0.28 | 0.7795 |

LP5282-P, paraprobiotic derived from *Lactiplantibacillus plantarum* LRCC5282; n, number of participants included in the per-protocol analysis from each high-diversity subgroup.

Data are presented as mean ± SEM. P-values represent between-group comparisons, with P < 0.05 considered statistically significant.

**Supplementary Table 6.** Hematology and serum chemistry parameters in the low-diversity subgroup from screening to week 12

| Contents | Placebo (Low-diversity, n = 26) | LP5282-P (Low-diversity, n = 24) | P-value |
| --- | --- | --- | --- |
| Albumin, g/dL | |  |  |
| - visit 1 (screening) | 4.59 ± 0.06 | 4.68 ± 0.06 | 0.3095 |
| - visit 4 (12-wks) | 4.65 ± 0.06 | 4.65 ± 0.05 | 0.9999 |
| - changes from baseline | 0.06 ± 0.04 | -0.03 ± 0.03 | 0.0908 |
| Alkaline phosphatase (ALP), IU/L | |  |  |
| - visit 1 (screening) | 59.27 ± 3.23 | 60.83 ± 3.48 | 0.7430 |
| - visit 4 (12-wks) | 64.73 ± 3.68 | 62.46 ± 4.22 | 0.6855 |
| - changes from baseline | 5.46 ± 1.76 | 1.63 ± 1.95 | 0.1487 |
| Alanine aminotransferase (ALT), IU/L | |  |  |
| - visit 1 (screening) | 21.35 ± 2.70 | 25.50 ± 2.87 | 0.2970 |
| - visit 4 (12-wks) | 22.31 ± 3.09 | 23.63 ± 2.26 | 0.7357 |
| - changes from baseline | 0.96 ± 1.38 | -1.88 ± 2.40 | 0.3007 |
| Aspartate aminotransferase (AST), IU/L | |  |  |
| - visit 1 (screening) | 24.04 ± 1.76 | 25.33 ± 2.68 | 0.6836 |
| - visit 4 (12-wks) | 23.77 ± 1.95 | 23.67 ± 1.32 | 0.9659 |
| - changes from baseline | -0.27 ± 0.97 | -1.67 ± 2.04 | 0.5287 |
| Basophil, % | |  |  |
| - visit 1 (screening) | 0.72 ± 0.08 | 0.54 ± 0.04 | 0.0601 |
| - visit 4 (12-wks) | 0.64 ± 0.08 | 0.57 ± 0.05 | 0.4635 |
| - changes from baseline | -0.08 ± 0.05 | 0.03 ± 0.03 | 0.0599 |
| Blood urea nitrogen (BUN), mg/dL | |  |  |
| - visit 1 (screening) | 12.28 ± 0.51 | 13.58 ± 0.75 | 0.1560 |
| - visit 4 (12-wks) | 11.92 ± 0.72 | 13.20 ± 0.63 | 0.1922 |
| - changes from baseline | -0.36 ± 0.67 | -0.38 ± 0.46 | 0.9831 |
| Calcium (Ca), mmol/L | |  |  |
| - visit 1 (screening) | 9.35 ± 0.07 | 9.45 ± 0.08 | 0.3383 |
| - visit 4 (12-wks) | 9.43 ± 0.06 | 9.54 ± 0.07 | 0.2526 |
| - changes from baseline | 0.08 ± 0.04 | 0.09 ± 0.06 | 0.8878 |
| Creatine kinase (CK), IU/L | |  |  |
| - visit 1 (screening) | 170.82 ± 23.80 | 165.33 ± 25.41 | 0.8746 |
| - visit 4 (12-wks) | 148.24 ± 26.18 | 152.37 ± 27.73 | 0.9156 |
| - changes from baseline | -22.62 ± 30.69 | -13.04 ± 25.86 | 0.8140 |
| Chloride (Cl), mmol/L | |  |  |
| - visit 1 (screening) | 104.01 ± 0.29 | 104.25 ± 0.41 | 0.6837 |
| - visit 4 (12-wks) | 103.74 ± 0.38 | 104.42 ± 0.48 | 0.2682 |
| - changes from baseline | -0.29 ± 0.37 | 0.20 ± 0.49 | 0.4265 |
| Creatinine, mg/dL | |  |  |
| - visit 1 (screening) | 0.76 ± 0.03 | 0.82 ± 0.04 | 0.2368 |
| - visit 4 (12-wks) | 0.75 ± 0.04 | 0.84 ± 0.03 | 0.0750 |
| - changes from baseline | -0.01 ± 0.02 | 0.03 ± 0.02 | 0.2108 |
| Eosinophil, % | |  |  |
| - visit 1 (screening) | 2.75 ± 0.47 | 1.86 ± 0.32 | 0.1328 |
| - visit 4 (12-wks) | 2.54 ± 0.36 | 1.74 ± 0.25 | 0.0817 |
| - changes from baseline | -0.21 ± 0.29 | -0.12 ± 0.14 | 0.7874 |
| Glucose, mg/dL | |  |  |
| - visit 1 (screening) | 100.37 ± 2.05 | 98.17 ± 2.32 | 0.4985 |
| - visit 4 (12-wks) | 96.54 ± 2.07 | 97.46 ± 2.08 | 0.7553 |
| - changes from baseline | -3.73 ± 2.10 | -0.71 ± 1.56 | 0.2597 |
| γ-glutamyl transferase (GTP), IU/L | |  |  |
| - visit 1 (screening) | 29.62 ± 4.67 | 27.71 ± 2.15 | 0.7200 |
| - visit 4 (12-wks) | 29.31 ± 5.49 | 27.96 ± 2.58 | 0.8272 |
| - changes from baseline | -0.31 ± 1.86 | 0.79 ± 1.65 | 0.6623 |
| Hemoglobin (Hb), g/dL | |  |  |
| - visit 1 (screening) | 14.08 ± 0.26 | 14.44 ± 0.24 | 0.3228 |
| - visit 4 (12-wks) | 14.10 ± 0.26 | 14.59 ± 0.23 | 0.1585 |
| - changes from baseline | 0.02 ± 0.15 | 0.15 ± 0.12 | 0.4777 |
| Hematocrit (Hct), % | |  |  |
| - visit 1 (screening) | 42.61 ± 0.67 | 43.55 ± 0.62 | 0.3122 |
| - visit 4 (12-wks) | 42.45 ± 0.65 | 43.99 ± 0.64 | 0.0991 |
| - changes from baseline | -0.16 ± 0.41 | 0.45 ± 0.42 | 0.3063 |
| Potassium (K), mmol/L | |  |  |
| - visit 1 (screening) | 4.22 ± 0.06 | 4.32 ± 0.05 | 0.2612 |
| - visit 4 (12-wks) | 4.24 ± 0.06 | 4.25 ± 0.06 | 0.8110 |
| - changes from baseline | 0.01 ± 0.06 | -0.06 ± 0.06 | 0.4065 |
| Lymphocyte, % | |  |  |
| - visit 1 (screening) | 35.57 ± 1.34 | 35.20 ± 1.80 | 0.8684 |
| - visit 4 (12-wks) | 33.11 ± 1.21 | 35.65 ± 1.87 | 0.2508 |
| - changes from baseline | -2.46 ± 1.23 | 0.46 ± 1.35 | 0.1157 |
| Mean corpuscular volume (MCV), fL | |  |  |
| - visit 1 (screening) | 91.17 ± 0.81 | 88.52 ± 0.86 | 0.3897 |
| - visit 4 (12-wks) | 90.86 ± 0.80 | 88.67 ± 0.88 | 0.4466 |
| - changes from baseline | -0.32 ± 0.33 | 0.15 ± 0.33 | 0.8266 |
| Monocyte, % | |  |  |
| - visit 1 (screening) | 7.17 ± 0.28 | 7.07 ± 0.34 | 0.8158 |
| - visit 4 (12-wks) | 7.28 ± 0.42 | 7.45 ± 0.46 | 0.7818 |
| - changes from baseline | 0.10 ± 0.34 | 0.38 ± 0.27 | 0.5355 |
| Sodium (Na), mmol/L | |  |  |
| - visit 1 (screening) | 139.83 ± 0.39 | 140.34 ± 0.50 | 0.3768 |
| - visit 4 (12-wks) | 140.16 ± 0.44 | 140.52 ± 0.38 | 0.5286 |
| - changes from baseline | 0.35 ± 0.39 | 0.16 ± 0.46 | 0.7501 |
| Neutrophil, % | |  |  |
| - visit 1 (screening) | 53.73 ± 1.42 | 55.29 ± 1.96 | 0.5175 |
| - visit 4 (12-wks) | 56.32 ± 1.43 | 54.53 ± 2.07 | 0.4719 |
| - changes from baseline | 2.60 ± 1.37 | -0.76 ± 1.53 | 0.1066 |
| Platelet (PLT), 10^3^/μL | |  |  |
| - visit 1 (screening) | 276.32 ± 11.87 | 270.04 ± 11.86 | 0.7093 |
| - visit 4 (12-wks) | 277.63 ± 11.69 | 276.42 ± 13.97 | 0.9492 |
| - changes from baseline | 1.23 ± 4.71 | 6.38 ± 10.72 | 0.6538 |
| Protein, g/dL | |  |  |
| - visit 1 (screening) | 7.30 ± 0.08 | 7.50 ± 0.07 | 0.0733 |
| - visit 4 (12-wks) | 7.44 ± 0.07 | 7.49 ± 0.07 | 0.5454 |
| - changes from baseline | 0.13 ± 0.07 | -0.00 ± 0.05 | 0.1138 |
| Red blood cell (RBC), 10^6^/μL | |  |  |
| - visit 1 (screening) | 4.69 ± 0.09 | 4.93 ± 0.09 | 0.0537 |
| - visit 4 (12-wks) | 4.68 ± 0.08 | 4.97 ± 0.09 | 0.0164 |
| - changes from baseline | -0.01 ± 0.04 | 0.04 ± 0.04 | 0.3922 |
| Total bilirubin, g/dL | |  |  |
| - visit 1 (screening) | 0.64 ± 0.04 | 0.73 ± 0.08 | 0.3242 |
| - visit 4 (12-wks) | 0.70 ± 0.04 | 0.75 ± 0.06 | 0.5539 |
| - changes from baseline | 0.06 ± 0.04 | 0.02 ± 0.07 | 0.5615 |
| White blood cell (WBC), 10^3^/μL | |  |  |
| - visit 1 (screening) | 6.05 ± 0.28 | 5.77 ± 0.20 | 0.4272 |
| - visit 4 (12-wks) | 6.20 ± 0.23 | 5.75 ± 0.23 | 0.1756 |
| - changes from baseline | 0.15 ± 0.16 | -0.02 ± 0.15 | 0.4538 |

LP5282-P, paraprobiotic derived from *Lactiplantibacillus plantarum* LRCC5282; n, number of participants included in the per-protocol analysis from each low-diversity subgroup.

Data are presented as mean ± SEM. P-values represent between-group comparisons, with P < 0.05 considered statistically significant.

**Supplementary Table 7.** Changes in anthropometric, body composition, and metabolic parameters at baseline and week 12 in the high-diversity subgroup

| Parameters | Placebo (High-diversity, n = 26) | LP5282-P (High-diversity, n = 25) | P-value |
| --- | --- | --- | --- |
| Body weight (kg) |  |  |  |
| - visit 2 (baseline) | 72.38 ± 1.61 | 69.72 ± 1.06 | 0.1756 |
| - visit 4 (after 12 weeks) | 71.13 ± 1.70 | 69.12 ± 1.09 | 0.3270 |
| - changes from baseline | -1.25 ± 0.36 | -0.60 ± 0.23 | 0.1312 |
| BMI (kg/m^2^) |  |  |  |
| - visit 2 (baseline) | 27.43 ± 0.38 | 26.47 ± 0.35 | 0.0710 |
| - visit 4 (after 12 weeks) | 26.89 ± 0.28 | 26.25 ± 0.23 | 0.0873 |
| - changes from baseline | -0.54 ± 0.12^a^ | -0.22 ± 0.07^b^ | 0.0283 |
| DXA (fat mass, g) |  |  |  |
| - visit 2 (baseline) | 25,983.27 ± 953.64 | 25,947.37 ± 782.33 | 0.9772 |
| - visit 4 (after 12 weeks) | 25,252.36 ± 827.13 | 25,669.55 ± 785.17 | 0.7170 |
| - changes from baseline | -730.24 ± 313.23 | -278.14 ± 235.50 | 0.2571 |
| DXA (fat mass, %) |  |  |  |
| - visit 2 (baseline) | 38.03 ± 1.45 | 38.62 ± 1.17 | 0.7529 |
| - visit 4 (after 12 weeks) | 37.63 ± 1.36 | 38.61 ± 1.08 | 0.5867 |
| - changes from baseline | -0.40 ± 0.31 | -0.00 ± 0.29 | 0.3539 |
| DXA (lean, g) |  |  |  |
| - visit 2 (baseline) | 42,926.53 ± 8,679.26 | 41,483.81 ± 6,064.58 | 0.4961 |
| - visit 4 (after 12 weeks) | 42,536.39 ± 1,720.59 | 41,001.57 ± 1,161.18 | 0.4663 |
| - changes from baseline | -390.0 ± 193.62 | -482.54 ± 225.04 | 0.7560 |
| Total Cholesterol (mg/dL) |  |  |  |
| - visit 2 (baseline) | 207.04 ± 6.07 | 192.81 ± 7.30 | 0.1388 |
| - visit 4 (after 12 weeks) | 198.68 ± 6.10 | 193.57 ± 7.88 | 0.6097 |
| - changes from baseline | -8.36 ± 4.80 | 0.76 ± 6.52 | 0.2612 |
| HDL-Cholesterol (mg/dL) |  |  |  |
| - visit 2 (baseline) | 55.12 ± 2.15 | 54.36 ± 2.14 | 0.8047 |
| - visit 4 (after 12 weeks) | 55.81 ± 2.64 | 53.20 ± 2.04 | 0.4408 |
| - changes from baseline | 0.69 ± 1.09 | -1.16 ± 1.33 | 0.2846 |
| LDL-Cholesterol (mg/dL) |  |  |  |
| - visit 2 (baseline) | 128.83 ± 6.16 | 116.64 ± 6.98 | 0.1951 |
| - visit 4 (after 12 weeks) | 124.58 ± 5.71 | 114.36 ± 9.17 | 0.3428 |
| - changes from baseline | -4.27 ± 4.49 | -2.32 ± 8.07 | 0.8320 |
| Triglycerides (mg/dL) |  |  |  |
| - visit 2 (baseline) | 134.82 ± 13.10 | 124.77 ± 83.58 | 0.6328 |
| - visit 4 (after 12 weeks) | 138.86 ± 12.43 | 127.65 ± 20.98 | 0.6460 |
| - changes from baseline | 3.96 ± 13.31 | 2.96 ± 9.46 | 0.9517 |
| hs-CRP (mg/dL) |  |  |  |
| - visit 2 (baseline) | 3.21 ± 1.15 | 1.87 ± 0.68 | 0.3241 |
| - visit 4 (after 12 weeks) | 4.54 ± 1.71 | 1.53 ± 0.46 | 0.1021 |
| - changes from baseline | 1.33 ± 1.27 | -0.34 ± 0.49 | 0.2321 |
| Adiponectin (μg/mL) |  |  |  |
| - visit 2 (baseline) | 5.61 ± 0.57 | 5.87 ± 0.39 | 0.7052 |
| - visit 4 (after 12 weeks) | 5.30 ± 0.55 | 5.68 ± 0.37 | 0.5075 |
| - changes from baseline | -0.31 ± 0.29 | -0.19 ± 0.25 | 0.6507 |
| Leptin (ng/mL) |  |  |  |
| - visit 2 (baseline) | 15.23 ± 1.80 | 13.07 ± 1.14 | 0.2190 |
| - visit 4 (after 12 weeks) | 13.96 ± 1.66 | 12.31 ± 1.40 | 0.4531 |
| - changes from baseline | -1.27 ± 1.19 | -0.71 ± 0.87 | 0.4832 |
| Visceral fat area (cm²) |  |  |  |
| - visit 2 (baseline) | 116.92 ± 7.33 | 112.27 ± 8.37 | 0.6676 |
| - visit 4 (after 12 weeks) | 104.36 ± 7.00 | 110.58 ± 8.34 | 0.5706 |
| - changes from baseline | -12.60 ± 5.02 | -1.61 ± 6.16 | 0.1714 |
| Subcutaneous fat area (cm²) |  |  |  |
| - visit 2 (baseline) | 260.74 ± 13.36 | 254.75 ± 13.45 | 0.7509 |
| - visit 4 (after 12 weeks) | 256.21 ± 13.76 | 247.53 ± 11.98 | 0.3006 |
| - changes from baseline | -4.52 ± 5.48 | -7.22 ± 9.62 | 0.1294 |
| Total abdominal fat area (cm²) |  |  |  |
| - visit 2 (baseline) | 377.76 ± 15.39 | 366.82 ± 15.70 | 0.6239 |
| - visit 4 (after 12 weeks) | 360.63 ± 14.70 | 353.71 ± 13.58 | 0.5216 |
| - changes from baseline | -17.12 ± 8.91 | -13.11 ± 10.03 | 0.8756 |
| Ratio of visceral/subcutaneous |  |  |  |
| - visit 2 (baseline) | 0.48 ± 0.04 | 0.48 ± 0.05 | 0.9187 |
| - visit 4 (after 12 weeks) | 0.44 ± 0.04 | 0.51 ± 0.05 | 0.3520 |
| - changes from baseline | -0.04 ± 0.02^a^ | 0.03 ± 0.03^b^ | 0.0397 |

BMI, body mass index; DXA, dual-energy X-ray absorptiometry; HDL, high-density lipoprotein; LDL, low-density lipoprotein; hs-CRP, high-sensitivity C-reactive protein; LP5282-P, paraprobiotic derived from *Lactiplantibacillus plantarum* LRCC5282; n, number of participants included in the per-protocol analysis from each high-diversity subgroup.

Data are presented as mean ± SEM. Different superscript letters (a, b) indicate statistically significant differences between groups (P < 0.05). P-values represent between-group comparisons, with P < 0.05 considered statistically significant.

**Supplementary Table 8.** Physical activity and sedentary behavior at baseline, week 6, and week 12 according to intervention and baseline gut microbial diversity

|  | Parameters | Placebo (High-diversity, n = 26) | LP5282-P (High-diversity, n = 25) | Placebo (Low-diversity, n = 26) | LP5282-P (Low-diversity, n = 24) | P-value |
| --- | --- | --- | --- | --- | --- | --- |
| work-related | vigorous (min/week) |  |  |  |  |  |
|  | - visit 2 (baseline) | 5.77 ± 4.12 | 57.60 ± 39.11 | 21.92 ± 14.66 | 22.92 ± 16.46 | 0.9240 |
|  | - visit 3 (after 6 weeks) | 77.31 ± 69.10 | 102.00 ± 73.85 | 70.38 ± 32.65 | 40.00 ± 26.46 | 0.5543 |
|  | - visit 4 (after 12 weeks) | 32.31 ± 22.63 | 56.80 ± 38.19 | 72.69 ± 57.36 | 25.42 ± 13.69 | 0.7077 |
|  | moderate (min/week) |  |  |  |  |  |
|  | - visit 2 (baseline) | 237.31 ±109.27 | 221.40 ± 91.39 | 242.70 ± 116.05 | 249.17 ± 97.01 | 0.9588 |
|  | - visit 3 (after 6 weeks) | 333.85 ± 114.11 | 155.60 ± 89.06 | 217.31 ± 96.20 | 298.75 ± 103.08 | 0.3074 |
|  | - visit 4 (after 12 weeks) | 283.46 ± 92.65 | 122.80 ± 47.93 | 264.23 ± 84.40 | 286.88 ± 82.63 | 0.1603 |
| sports-related | vigorous (min/week) |  |  |  |  |  |
|  | - visit 2 (baseline) | 48.85 ± 19.80 | 36.00 ± 18.95 | 85.77 ± 26.91 | 43.33 ± 20.17 | 0.2578 |
|  | - visit 3 (after 6 weeks) | 572.31 ± 229.07 | 227.20 ± 99.67 | 867.70 ± 264.54 | 273.33 ± 132.31 | 0.2998 |
|  | - visit 4 (after 12 weeks) | 535.39 ± 168.44 | 358.40 ± 117.31 | 556.92 ± 228.81 | 613.33 ± 202.69 | 0.8567 |
|  | moderate (min/week) |  |  |  |  |  |
|  | - visit 2 (baseline) | 141.54 ± 27.59 | 150.80 ± 38.41 | 150.19 ±30.57 | 140.00 ± 43.62 | 0.8546 |
|  | - visit 3 (after 6 weeks) | 152.89 ± 30.57 | 207.60 ± 45.00 | 145.39 ± 24.18 | 121.88 ± 18.92 | 0.8913 |
|  | - visit 4 (after 12 weeks) | 244.23 ± 86.39 | 206.00 ± 47.67 | 153.46 ± 27.33 | 124.58 ± 26.43 | 0.8392 |
| walking-cycling | moderate (min/week) |  |  |  |  |  |
|  | - visit 2 (baseline) | 219.04 ± 51.53 | 181.20 ± 33.48 | 226.73 ± 44.44 | 203.54 ± 44.94 | 0.9546 |
|  | - visit 3 (after 6 weeks) | 236.73 ± 54.56 | 189.60 ± 36.09 | 191.92 ± 43.10 | 185.42 ± 35.23 | 0.8928 |
|  | - visit 4 (after 12 weeks) | 285.77 ± 59.26 | 250.60 ± 54.50 | 191.54 ± 41.25 | 167.50 ± 27.33 | 0.6297 |
| sedentary  time | moderate (min/day) |  |  |  |  |  |
|  | - visit 2 (baseline) | 432.69 ± 47.54 | 426.80 ± 51.04 | 489.23 ± 42.74 | 371.25 ± 41.25 | 0.3244 |
|  | - visit 3 (after 6 weeks) | 432.69 ± 42.17 | 460.80 ± 45.39 | 426.92 ± 44.72 | 431.25 ± 46.77 | 0.9697 |
|  | - visit 4 (after 12 weeks) | 477.69 ± 45.33 | 483.60 ± 43.03 | 414.23 ± 38.09 | 405.00 ± 42.35 | 0.5800 |

LP5282-P, paraprobiotic derived from *Lactiplantibacillus plantarum* LRCC5282; n, number of participants included in the per-protocol analysis from each subgroup.

Data are presented as mean ± SEM. Work-related, sports/leisure-related, and transport-related activities are expressed as min/week, calculated by multiplying the number of active days per week by the reported duration per active day. Sedentary time is expressed as min/day. P-values represent overall comparisons among the four groups at each visit using Welch’s one-way ANOVA. P < 0.05 was considered statistically significant.

**Supplementary Table 9.** MET-based physical activity and sedentary behavior at baseline, week 6, and week 12 according to intervention and baseline gut microbial diversity

|  | Parameters | Placebo (High-diversity, n = 26) | LP5282-P (High-diversity, n = 25) | Placebo (Low-diversity, n = 26) | LP5282-P (Low-diversity, n = 24) | P-value |
| --- | --- | --- | --- | --- | --- | --- |
| work-related | vigorous (MET-min/week) |  |  |  |  |  |
|  | - visit 2 (baseline) | 46.15 ± 37.70 | 460.81 ± 312.91 | 175.43 ± 117.22 | 183.32 ± 131.74 | 0.3454 |
|  | - visit 3 (after 6 weeks) | 618.54 ± 552.79 | 816.00 ± 590.81 | 563.08 ± 261.20 | 320.00 ± 211.66 | 0.5543 |
|  | - visit 4 (after 12 weeks) | 258.46 ± 181.04 | 454.40 ± 305.50 | 581.54 ± 458.89 | 203.33 ± 109.49 | 0.7077 |
|  | moderate (MET-min/week) |  |  |  |  |  |
|  | - visit 2 (baseline) | 949.23 ± 437.08 | 885.60 ± 365.55 | 970.77 ± 464.22 | 996.67 ± 388.02 | 0.9588 |
|  | - visit 3 (after 6 weeks) | 1,335.38 ± 456.42 | 622.40 ± 356.23 | 869.23 ± 384.80 | 1,195.00 ± 412.32 | 0.3074 |
|  | - visit 4 (after 12 weeks) | 1,133.85 ± 370.62 | 491.20 ± 191.70 | 1,056.92 ± 337.58 | 1,147.50 ± 333.52 | 0.1603 |
| sports-related | vigorous (MET-min/week) |  |  |  |  |  |
|  | - visit 2 (baseline) | 393.77 ± 158.41 | 288.00 ± 151.58 | 686.15 ± 215.27 | 346.67 ± 161.35 | 0.2578 |
|  | - visit 3 (after 6 weeks) | 572.31 ± 229.07 | 227.20 ± 99.67 | 867.69 ± 264.54 | 273.33 ± 132.31 | 0.2998 |
|  | - visit 4 (after 12 weeks) | 535.39 ± 168.44 | 358.40 ± 117.31 | 556.92 ± 228.81 | 613.33 ± 202.69 | 0.8567 |
|  | moderate (MET-min/week) |  |  |  |  |  |
|  | - visit 2 (baseline) | 556.15 ± 110.38 | 603.20 ± 153.66 | 600.77 ± 122.28 | 560.00 ± 174.49 | 0.8546 |
|  | - visit 3 (after 6 weeks) | 611.54 ± 122.29 | 830.40 ± 179.99 | 581.54 ± 96.72 | 487.50 ± 75.67 | 0.8913 |
|  | - visit 4 (after 12 weeks) | 976.92 ± 345.57 | 824.00 ± 190.66 | 613.85 ± 109.33 | 498.33 ± 105.72 | 0.8392 |
| walking-cycling | (MET-min/week) |  |  |  |  |  |
|  | - visit 2 (baseline) | 876.15 ± 206.13 | 724.80 ± 133.93 | 906.92 ± 177.76 | 814.17 ± 179.78 | 0.9546 |
|  | - visit 3 (after 6 weeks) | 946.92 ± 218.25 | 758.40 ± 144.34 | 767.69 ± 172.41 | 741.67 ± 140.92 | 0.8928 |
|  | - visit 4 (after 12 weeks) | 1,143.08 ± 237.04 | 1,002.40 ± 218.01 | 766.15 ± 165.01 | 670.00 ± 109.33 | 0.6297 |
| sedentary  time | (min/day) |  |  |  |  |  |
|  | - visit 2 (baseline) | 432.69 ± 47.54 | 426.80 ± 51.04 | 489.23 ± 42.74 | 371.25 ± 41.25 | 0.3244 |
|  | - visit 3 (after 6 weeks) | 432.69 ± 42.17 | 460.80 ± 45.39 | 426.92 ± 44.72 | 431.25 ± 46.77 | 0.9697 |
|  | - visit 4 (after 12 weeks) | 477.69 ± 45.33 | 483.60 ± 43.03 | 414.23 ± 38.09 | 405.00 ± 42.35 | 0.5800 |
| Total physical activity | (MET-min/week) |  |  |  |  |  |
|  | - visit 2 (baseline) | 2,828.46 ± 528.92 | 2,962.40 ± 598.41 | 3,340.00 ± 611.59 | 2,900.83 ± 675.43 | 0.8381 |
|  | - visit 3 (after 6 weeks) | 4,084.62 ± 791.53 | 3,254.40 ± 809.41 | 3,649.23 ± 812.92 | 2,957.50 ± 620.91 | 0.6028 |
|  | - visit 4 (after 12 weeks) | 4,047.69 ± 562.50 | 3,130.40 ± 479.39 | 3,575.38 ± 760.98 | 3,099.17 ± 505.21 | 0.6560 |

LP5282-P, paraprobiotic derived from *Lactiplantibacillus plantarum* LRCC5282; MET, metabolic equivalent of task; n, number of participants included in the per-protocol analysis from each subgroup.

Data are presented as mean ± SEM. Physical activity is expressed as MET-min/week, calculated using 8 METs for vigorous activity and 4 METs for moderate activity and transport-related walking or cycling. Sedentary time is expressed as min/day and was excluded from total physical activity. P-values represent overall comparisons among the four groups at each visit using Welch’s one-way ANOVA. P < 0.05 was considered statistically significant.

**Supplementary Table 10.** Subgroup treatment comparisons with physical activity included as a covariate.

|  | Parameters | Placebo (High-diversity, n = 26) | LP5282-P (High-diversity, n = 25) | Placebo (Low-diversity, n = 26) | LP5282-P (Low-diversity, n = 24) |
| --- | --- | --- | --- | --- | --- |
| Adjusted for  baseline covariates | Clinical outcomes |  |  |  |  |
|  | - ΔBody weight (kg) | -1.25 ± 0.36^ab^ | -0.60 ± 0.23^b^ | -0.95 ± 0.33^ab^ | -1.98 ± 0.39^a^ |
|  | - ΔBMI (kg/m²) | -0.54 ± 0.12^ab^ | -0.22 ± 0.07^b^ | -0.31 ± 0.13^ab^ | -0.74 ± 0.15^a^ |
|  | - ΔBody fat mass (g) | -730.24 ± 313.23 | -278.14 ± 235.50 | -410.11 ± 255.13 | -890.92 ± 400.24 |
|  | - ΔLeptin (ng/mL) | -1.27 ± 1.19^b^ | -0.71 ± 0.87^b^ | -0.24 ± 0.53^b^ | -2.07 ± 0.71^a^ |
|  | Genus relative abundance (Δ%) |  |  |  |  |
|  | *- Akkermansia* | -0.03 ± 0.08 | 0.06 ± 0.08 | -0.77 ± 0.73 | 1.84 ± 0.92 |
|  | *- Eubacterium* | 0.00 ± 0.02 | 0.02 ± 0.01 | 0.01 ± 0.01 | 0.05 ± 0.03 |
|  | *- Faecalibacterium* | 0.07 ± 0.22^a^ | 0.00 ± 0.19^a^ | -2.51 ± 1.51^a^ | 5.40 ± 2.21^b^ |
|  | *- Oscillibacter* | -0.03 ± 0.04^a^ | 0.07 ± 0.09^a^ | 0.00 ± 0.04^a^ | 0.37 ± 0.07^b^ |
|  | *- Christensenella* | -0.65 ± 0.13^a^ | 0.01 ± 0.19^bc^ | -0.06 ± 0.09^b^ | 0.68 ± 0.32^c^ |
|  | *- Alistipes* | -0.01 ± 0.02 | 0.00 ± 0.02 | 0.02 ± 0.02 | 0.06 ± 0.03 |
|  | *- Bacteroides* | 0.16 ± 0.05 | 0.17 ± 0.08 | -0.01 ± 0.09 | 0.16 ± 0.07 |
| Additionally adjusted for  physical activity | Clinical outcomes |  |  |  |  |
|  | - ΔBody weight (kg) | -1.25 ± 0.36^ab^ | -0.60 ± 0.23^b^ | -0.95 ± 0.33^ab^ | -1.98 ± 0.39^a^ |
|  | - ΔBMI (kg/m²) | -0.54 ± 0.12^ab^ | -0.22 ± 0.07^b^ | -0.31 ± 0.13**^b^** | -0.74 ± 0.15^a^ |
|  | - ΔBody fat mass (g) | -730.24 ± 313.23 | -278.14 ± 235.50 | -410.11 ± 255.13 | -890.92 ± 400.24 |
|  | - ΔLeptin (ng/mL) | -1.27 ± 1.19^b^ | -0.71 ± 0.87^b^ | -0.24 ± 0.53^b^ | -2.07 ± 0.71^a^ |
|  | Genus relative abundance (Δ%) |  |  |  |  |
|  | *- Akkermansia* | -0.03 ± 0.08**^ab^** | 0.06 ± 0.08**^ab^** | -0.77 ± 0.73**^a^** | 1.84 ± 0.92**^b^** |
|  | *- Eubacterium* | 0.00 ± 0.02 | 0.02 ± 0.01 | 0.01 ± 0.01 | 0.05 ± 0.03 |
|  | *- Faecalibacterium* | 0.07 ± 0.22^a^ | 0.00 ± 0.19^a^ | -2.51 ± 1.51^a^ | 5.40 ± 2.21^b^ |
|  | *- Oscillibacter* | -0.03 ± 0.04^a^ | 0.07 ± 0.09^a^ | 0.00 ± 0.04^a^ | 0.37 ± 0.07^b^ |
|  | *- Christensenella* | -0.65 ± 0.13^a^ | 0.01 ± 0.19**^b^** | -0.06 ± 0.09**^ab^** | 0.68 ± 0.32^c^ |
|  | *- Alistipes* | -0.01 ± 0.02 | 0.00 ± 0.02 | 0.02 ± 0.02 | 0.06 ± 0.03 |
|  | *- Bacteroides* | 0.16 ± 0.05 | 0.17 ± 0.08 | -0.01 ± 0.09 | 0.16 ± 0.07 |

LP5282-P, paraprobiotic derived from *Lactiplantibacillus plantarum* LRCC5282; MET, metabolic equivalent of task; Δ, change from baseline; n, number of participants included in the per-protocol analysis from each subgroup.
Data are presented as mean ± SEM of the change from baseline to week 12. The upper panel was adjusted for baseline covariates, and the lower panel was additionally adjusted for physical activity, comprising baseline total physical activity (MET-minutes/week), change in total physical activity from baseline to week 12, and baseline sedentary time. Significance was assessed using linear regression with pairwise contrasts among the four subgroups and the Benjamini–Hochberg false discovery rate correction. Different superscript letters indicate statistically significant differences among the four subgroups (P < 0.05).

**Supplementary Figure 1.** Summed total physical activity at weeks 6 and 12 according to intervention and baseline gut microbial diversity.

PL-H, high-diversity placebo subgroup; LP-H, high-diversity LP5282-P subgroup; PL-L, low-diversity placebo subgroup; LP-L, low-diversity LP5282-P subgroup; LP5282-P, paraprobiotic derived from *Lactiplantibacillus plantarum* LRCC5282; MET, metabolic equivalent of task.

Summed total physical activity was calculated for each participant by summing the total physical activity measured at weeks 6 and 12. Each symbol represents an individual participant. Horizontal lines and error bars indicate the mean ± SEM. Differences among the four subgroups were evaluated using Welch’s one-way ANOVA followed by Dunnett’s T3 multiple comparisons test. ns, not significant.

**Supplementary Figure 2.** Individual-level changes in clinical and metabolic outcomes in diversity-stratified subgroups.

Change from baseline to week 12 in (a) body weight, (b) body mass index (BMI), (c) dual-energy X-ray absorptiometry (DXA)-derived total body fat mass, (d) total-cholesterol, (e) HDL-cholesterol, (f) LDL-cholesterol, (g) adiponectin, (h) leptin, (i) visceral fat area, and (j) total abdominal fat area across all four subgroups.

PL-H, high-diversity placebo subgroup; LP-H, high-diversity LP5282-P subgroup; PL-L, low-diversity placebo subgroup; LP-L, low-diversity LP5282-P subgroup; LP5282-P, paraprobiotic derived from *Lactiplantibacillus plantarum* LRCC5282; BMI, body mass index; DXA, dual-energy X-ray absorptiometry; HDL, high-density lipoprotein; LDL, low-density lipoprotein. Each line connects the baseline and week 12 values of an individual participant, and the value shown in each panel indicates the mean change ± SEM within the subgroup.
